# Assessing diversity in canopy architecture, photosynthesis, and water‐use efficiency in a cowpea magic population

**DOI:** 10.1002/fes3.236

**Published:** 2020-08-07

**Authors:** Anthony Digrado, Noah G. Mitchell, Christopher M. Montes, Paulina Dirvanskyte, Elizabeth A. Ainsworth

**Affiliations:** ^1^ Global Change and Photosynthesis Research Unit USDA ARS Urbana IL USA; ^2^ Department of Plant Biology University of Illinois at Urbana‐Champaign Urbana IL USA; ^3^ Institute for Genomic Biology University of Illinois at Urbana‐Champaign Urbana IL USA; ^4^ Oxford University Oxford UK

**Keywords:** breeding, canopy architecture, canopy photosynthesis, LAI, MAGIC, stem angle, WUE

## Abstract

Optimizing crops to improve light absorption and CO_2_ assimilation throughout the canopy is a proposed strategy to increase yield and meet the needs of a growing population by 2050. Globally, the greatest population increase is expected to occur in Sub‐Saharan Africa where large yield gaps currently persist; therefore, it is crucial to develop high‐yielding crops adapted to this region. In this study, we screened 50 cowpea (*Vigna unguiculata* (L.) Walp) genotypes from the multi‐parent advanced generation inter‐cross (MAGIC) population for canopy architectural traits, canopy photosynthesis, and water‐use efficiency using a canopy gas exchange chamber in order to improve our understanding of the relationships among those traits. Canopy architecture contributed to 38.6% of the variance observed in canopy photosynthesis. The results suggest that the light environment within the canopy was a limiting factor for canopy CO_2_ assimilation. Traits favoring greater exposure of leaf area to light such as the width of the canopy relative to the total leaf area were associated with greater canopy photosynthesis, especially in canopies with high biomass. Canopy water‐use efficiency was highly determined by canopy photosynthetic activity and therefore canopy architecture, which indicates that optimizing the canopy will also contribute to improving canopy water‐use efficiency. We discuss different breeding strategies for future programs aimed at the improvement of cowpea yield for the Sub‐Saharan African region. We show that breeding for high biomass will not optimize canopy CO_2_ assimilation and suggest that selection should include multiple canopy traits to improve light penetration.

## Graphical abstract

In this study, 50 cowpea (*Vigna unguiculata* (L.) Walp) genotypes from the multi‐parent advanced generation inter‐cross (MAGIC) population were screened for canopy architectural traits, canopy photosynthesis, and water‐use efficiency using a canopy gas exchange chamber in order to improve our understanding of the relationship among those traits. Traits favoring greater exposure of leaf area to light such as the width of the canopy relative to the total leaf area were associated with greater canopy photosynthesis, especially in canopies with high biomass. Canopy water‐use efficiency was highly determined by canopy photosynthetic activity and therefore canopy architecture, which indicates that optimizing the canopy will also contribute to improving canopy water‐use efficiency.

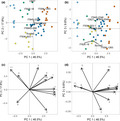

## 1 INTRODUCTION

Populations in most Sub‐Saharan African countries are expected to double by 2050 (FAO, 2017) further threatening food security in regions where approximately 23% of the population is undernourished (FAO, 2015). There are significant yield gaps in Sub‐Saharan African countries (van Ittersum et al., 2016) caused by complex constraints from abiotic, biotic, socioeconomic, and management factors (Waddington, Li, Dixon, Hyman, & de Vicente, 2010). The use of unimproved or unsuitable varieties of legumes contributes to production constraints and yield gaps in Sub‐Saharan Africa (Waddington et al., 2010). Therefore, it is crucial to identify strategies that sustainably enhance yield in crops that are widely grown by African farmers and contribute to food security.

Global cowpea (*Vigna unguiculata* (L.) Walp) production is estimated to be 4.5–6.5 million tons/year, with approximately 80% of production in West Africa. Nigeria, alone, is responsible for 45% of the world's cowpea production (Jayathilake et al., 2018; Langyintuo et al., 2003; Sprent, Odee, & Dakora, 2009). Although flowers and leaves are consumed in some regions, cowpea is mainly produced for beans, which provide a high‐quality plant protein source. Seeds contain 23%–32% protein, 50%–60% carbohydrate, and 1% fat (El Naim & Jabereldar, 2010; Jayathilake et al., 2018). Cowpea residues also serve as a high‐quality fodder for animals during the dry season in West Africa (Singh, Ajeigbe, Tarawali, Fernandez‐Rivera, & Abubakar, 2003; Tarawali, Okike, Kristjanson, Singh, & Thornton, 2005). In addition, inclusion of cowpea in the cropping system can contribute to restoration of soil fertility through the fixation of atmospheric nitrogen (Fatokun, Tarawali, Singh, Kormawa, & Tamò, 2002). Yet productivity of cowpea in Africa is low with average yields ranging 100–400 kg/ha (Kamara et al., 2017). For comparison with another dry bean, the average yield in the USA for soybean was 3,500 kg/ha in 2018 (USDA National Agricultural Statistics Service, 2019).

Enhancement of the photosynthetic process is often cited as a strategy to increase yield in crops (Bailey‐Serres, Parker, Ainsworth, Oldroyd, & Schroeder, 2019; Long, Zhu, Naidu, & Ort, 2006; Simkin, López‐Calcagno, & Raines, 2019; Weber & Bar‐Even, 2019; Wu, Hammer, Doherty, von Caemmerer, & Farquhar, 2019; Zhu, Long, & Ort, 2010). Among the different possible pathways to improve canopy photosynthesis, alteration of the canopy architecture has been shown to contribute to yield enhancement in different crops including soybean (Srinivasan, Kumar, & Long, 2016) and maize (Liu et al., 2015). Those results are indications that canopy architecture can still be optimized in a way that benefits yield. An optimized canopy ideally allows improved distribution of radiation and reduces excess light saturation maximizing canopy CO_2_ assimilation over the course of a day (Long et al., 2006; Sheehy & Mitchell, 2013). It should also optimize the size of the vegetative reservoir of nitrogen that is later relocated to the grains (Sheehy & Mitchell, 2013; Sinclair & Sheehy, 1999).

Water is a key driver of crop productivity (Mateos & Araus, 2016), and historically, breeding for greater plant biomass and seed production has inadvertently selected for increased stomatal conductance (Roche, 2015). However, the development of varieties with abundant water demands may be unfit for cultivation in Africa where access to fresh water, its distribution and/or its usage, is still challenging especially for smallholder farmers (Burney, Naylor, & Postel, 2013). Indeed, most of the crops in Sub‐Saharan Africa are currently rain‐fed (Burney et al., 2013) contributing to the large gap between the yields that are currently obtained and what could be potentially achieved under a scenario where water is non‐limiting (Rosa et al., 2018). For these reasons, optimization of water‐use efficiency is a key part of any strategy to develop new varieties for Africa.

The multi‐parent advanced generation inter‐cross (MAGIC) cowpea population has been developed by inter‐mating eight founder parent lines for several cycles (Huynh et al., 2018). The founders were genetically diverse and were selected based on their ability to produce high yield under drought conditions, along with other relevant agronomic traits (e.g., abiotic and biotic stress resistance and seed quality). To date, data assessing how canopy architecture affects canopy CO_2_ assimilation and water‐use efficiency in cowpea are scarce. As photosynthesis is an important factor determining the yield potential of a crop and its water consumption, we have investigated the association of canopy architectural traits and photosynthetic traits in 50 lines from the MAGIC cowpea population. We addressed the following questions: (a) what diversity exists in canopy architecture and canopy photosynthesis within the MAGIC germplasm collection; (b) how do canopy architectural features influence canopy photosynthesis; (c) which traits exert the most influence on cowpea water use efficiency (WUE)? Answers to these questions can inform efforts to develop cowpea cultivars with high yield potential and high water‐use efficiency.

## 2 MATERIALS AND METHODS

### 2.1 Cowpea genotypes and field design

Fifty lines from a cowpea MAGIC population (8 founder parents plus 42 recombinant inbred lines, Supporting information 1) were planted at the University of Illinois Energy Farm Facility in Urbana, IL (40.06°N, 88.21°W) on June 26th, 2019. Inbred lines with contrasting canopy architectural traits were selected based on previous phenotyping in Puerto Rico (data not shown). Each line was planted in a single 133.5 cm row oriented North‐South. Spacing between plants within a row was 3.8 cm (considered high‐density for cowpea) while the spacing between rows was 152.4 cm, which avoided competition for light between rows and maximized interception of incoming radiation within a single row. The cowpea lines were planted in a 5 × 10 grid with soybean planted in the periphery to limit potential border effects. Plants were occasionally watered during prolonged period of hot and dry days during the vegetative stage prior to phenotyping. No fertilization or pesticide treatments were applied. All measurements were performed when the genotypes had reached the reproductive developmental stage between R1 (early bloom) and R3 (early pod set).

### 2.2 Phenotyping

Three days prior to gas exchange measurements, cowpea lines were phenotyped for leaf length and width using a ruler (*n* = 5 leaves from different plants), greenness using the SPAD value of a chlorophyll‐meter (*n* = 5 leaves from different plants; SPAD‐502, Konica Minolta Sensing), number of nodes (*n* = 4), stem angle (*n* = 10) using a digital protractor smartphone application (Sensors multitool v1.3.2 for Android), canopy width using measurements of the width of the canopy at different position in the row (*n* = 5), and the height of the canopy (*n* = 5). Leaf measurements were performed on the last fully developed non‐senescing central leaflet at the fifth to seventh node down from the top of the canopy depending on cowpea line. After gas exchange measurements (described below), plants within the footprint of the canopy chamber were harvested. Leaves were separated from stems; total leaf area (LA) was measured with a leaf area meter (LI‐3100C Area Meter, Licor), stem length was measured for 4 plants per line; and then leaves and stems were dried separately in an oven at 60℃ for three weeks. Subsequently, samples were weighed for total leaf mass (g) and total shoot mass (g). The total biomass (g) was obtained from the sum of total leaf mass and total shoot mass. A parameter was introduced to describe the amount of leaf area exposed to solar irradiance (leaf area exposure) and was estimated as Canopy width (cm) ÷ Total LA. A high value for this parameter indicated a wide canopy that exposed a large proportion of its leaf area while a low value indicated a narrow, self‐shaded canopy. The LAI (leaf area index) was estimated as the total LA of harvested leaves (m^2^) ÷ enclosed canopy footprint (m^2^). The canopy footprint was calculated based on the averaged Canopy width (*n* = 5) on 1 m length. All raw values are provided in Supporting information 1.

### 2.3 Gas exchange measurements

Gas exchange measurements were made on two consecutive clear days 62 days after planting. Midday (between 11:00 and 15:00) leaf photosynthetic activity was measured in the field using a four portable gas exchange systems (Li‐6800, Licor) with an external light source (leaf net CO_2_ assimilation; *A*_leaf_) and a clear‐top chamber (*A*_leaf,CT_). Measurements (*n* = 4) were performed on a mature central leaflet fully exposed to the sun within each cowpea line. Cuvette conditions within the instrument were as follows: [CO_2_] = 420 μmol CO_2_ m^‐2^ s^‐1^, flow = 400 μmol m^‐2^ s^‐1^, and fan speed = 1,000 RPM. The relative humidity (RH), photosynthetic photon flux density (PPFD), and temperature (T) conditions inside the leaf chamber were set to match ambient conditions (RH = 73%–75% v/v, T = 31–33°C and when measurements were performed with the external light source PPFD = 1900 μmol m^‐2^ s^‐1^). Leaf intrinsic water‐use efficiency (iWUE_leaf_, μmol CO_2_ mol^‐1^ H_2_O) was estimated as *A*_leaf_ ÷ stomatal conductance (*g_s_*, mol H_2_O m^‐2^ s^‐1^). Immediately after the completion of leaf‐level gas exchange measurements (less than 5 min), canopy‐level photosynthetic activity was assessed once for each cowpea line using a closed‐system, portable canopy chamber.

The canopy chamber consisted of aluminum framing (1.2 × 1 × 1 m; h × l × w) covered with thin polycarbonate panels. Air inside the chamber was sampled at a 1 liter per minute and analyzed by a CO_2_/H_2_O gas analyzer (LI‐7000, Licor) for gas concentration measurements. Conditions inside the chamber were monitored by a temperature/relative humidity probe (HMP60, Vaisala), quantum sensor (LI‐190R, Licor), barometric pressure sensor (SB‐100, Apogee Instruments), and IR radiometer (SI‐121‐SS, Apogee Instruments). The cowpea canopy (17 ± 2 plants) was enclosed for 3 min inside the chamber in order to measure gas exchange. The canopy CO_2_ assimilation was calculated as a function of the rate of CO_2_ drawdown inside the chamber (dCO_2_/dt, μmol mol^‐1^s^‐1^). A quadratic regression was used to describe the CO_2_ depletion rate following the methods described by Pérez‐Priego, Testi, Orgaz, and Villalobos (2010):

$$\left[{\text{CO}}_{2}\right]=a+bt+ct^{2}$$

$$\frac{d{\text{CO}}_{2}}{\mathrm{dt}}=b+2ct$$

The quadratic regression method is usually preferred over a linear regression method to describe the rate of concentration change as the gradient of CO_2_ declines over time inside a closed chamber (Steduto, Çetinkökü, Albrizio, & Kanber, 2002). Canopy CO_2_ assimilation rate (*A*_canopy,ground_, μmol m^‐2^ s^‐1^) of the enclosed vegetation was then calculated following the equation published by Song and Zhu (2018):

$$A_{\text{canopy,flux}}=\frac{d{\text{CO}}_{2}}{\mathrm{dt}}.\frac{\text{PV}}{\text{SRT}}$$

where *V* (*m* ^3^) is the volume of the chamber, P (kPa) is the air pressure in the chamber, S (*m* ^2^) is the ground area that the canopy occupied, R is the universal gas constant (8.3 × 103 m^3^ kPa/mol K^‐1^), and T (*K*) is the air temperature in the chamber. In the same way, the canopy conductance was calculated based on the transpiration rate (E, mmol H_2_O m^‐2^ s^‐1^) in the canopy chamber, following a quadratic regression:

$\frac{\mathrm{dE}}{\mathrm{dt}}=b+2ct$

The canopy conductance (*g_C_*, mmol H_2_O m^‐2^ s^‐1^) was then estimated by the following equation based on Pérez‐Priego et al. (2010):

$g_{C}=\frac{\mathrm{dE}}{\mathrm{dt}}.\frac{\gamma }{\rho C_{P}}.\frac{\lambda }{\text{VPD}}$where γ is the psychometric constant (kPa/K), C_P_ the specific heat capacity of air at constant pressure (KJ kg^‐1^ K^‐1^), ρ the air density (kg/m^3^), λ is the specific heat of vaporization (MJ/kg), and VPD the vapor pressure deficit (kPa).

The intrinsic water‐use efficiency of cowpea (iWUE_canopy_, μmol CO_2_/mmol H_2_O) was calculated as *A*_canopy,ground_ ÷ *g_C_*. iWUE was also scaled to biomass by dividing iWUE_canopy_ by total biomass (iWUE_biomass_, g/mmol H_2_O m^‐2^ s^‐1^).

The first 8 s of logged measurements after canopy chamber closure was ignored in order to allow sufficient time for air mixing and stability inside the chamber. Then, canopy CO_2_ assimilation was calculated using a 30 s time window every 2 s. The median of *A*_canopy,ground_ values calculated this way for the first minute of measurement was used as the estimate for the canopy CO_2_ assimilation rate. In order to assess how efficiently CO_2_ was assimilated by the canopy relative to the total leaf area, *A*_canopy,ground_ was normalized by total LA (*A*_canopy,ground_ ÷ LA = *A*_canopy,LA_, μmol CO_2_ m^‐2^ s^‐1^). Canopy photosynthesis was measured once per cowpea line. The same canopy chamber was used for measurement of all cowpea lines.

In order to assess the impact of taking measurements on two days, we conducted the study by either analyzing the two days separately or by applying a corrective factor to gas exchange measurements based on the difference between the two medians. Because the results from the two approaches did not change the main conclusions, measurements from the two days were pooled together for subsequent analyses.

### 2.4 Statistical analysis

Pearson's correlation coefficient was calculated to assess correlations among all measured traits. General linear regression (GLM) models were then used to assess the portion of variance explained by individual traits. In the next stage, linear regression models including all canopy traits (with the exception of canopy‐level gas exchange measurements and LA/LAI because of their high correlation with total leaf mass) were tested for the prediction of the canopy CO_2_ assimilation (ground flux and LA‐based ground flux) and water‐use efficiency (canopy and biomass‐based). The best minimum adequate model was selected using a stepwise algorithm based on the Akaike's information criterion (AIC). The total variance explained by the models (*R* ^2^) was then decomposed to evaluate the relative variance explained by each predictor.

All traits related to canopy architecture (with the exception of LA and LAI) were entered as variables in a principal component analysis (PCA) based on a correlation matrix (i.e., appropriate method when variables have different scales) followed by hierarchical clustering on principle components using the Ward's criterion (HCPC). The average for all traits was calculated inside each defined cluster, and the Tukey's honest significant difference test (Tukey's HSD) was used to compare the means. In order to create diverse groups of cowpea lines with different canopy architectures of sufficient size and with a high homogeneity within each cluster, five clusters were defined with this analysis (inertia = 0.5). Similar results were obtained with fewer clusters (see Supporting information 4–6).

An ANCOVA was performed with the previously defined clusters in order to assess if the linear relationship between *A*_canopy,ground_ and *A*_canopy,LA_ was the same among clusters with constrasting architectures. In addition, linear regressions between canopy CO_2_ assimilation (ground flux and LA‐based ground flux) and all measured traits were performed within each cluster to assess which traits explained the most variance in canopy CO_2_ assimilation.

All statistical analyses were performed using RStudio version 1.1.453 (RStudio team, 2015), with the following R package: “FACTOMINER” (Lê, Josse, & Husson, 2008), “CAR” (Fox et al., 2016), and “RELAIMPO” (Groemping, 2006).

## 3 RESULTS

### 3.1 Variation in photosynthetic and canopy traits

**Figure 1:**
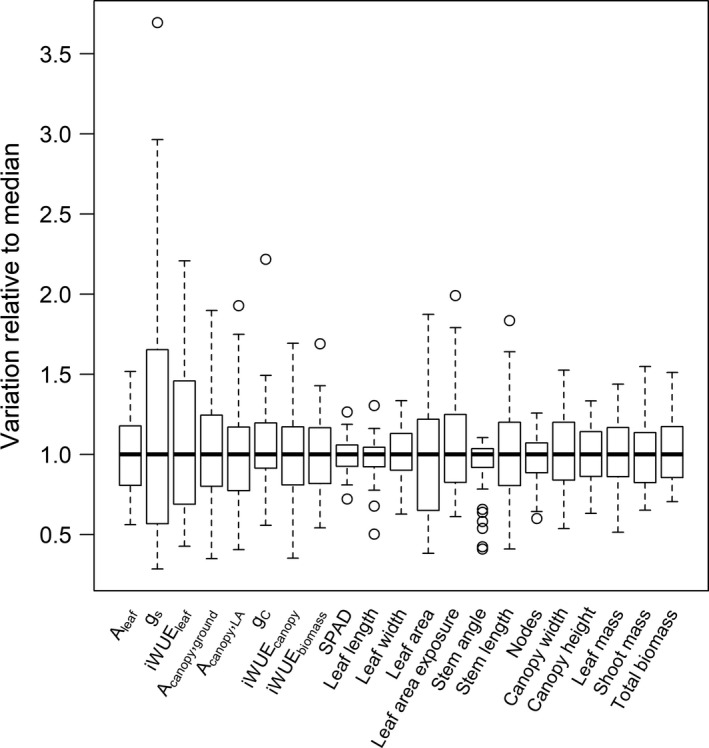
Boxplot showing the variability of phenotype traits measured for all cowpea genotypes (*n* = 50). In order to allow comparison of the variability among traits, measured values were normalized by dividing each value by the median value for that trait. The y axis, thus, represents the distribution of normalized values for each trait, where the normalized (N) value *j* for the trait *i* is equal to $N_{i,j}=\frac{x_{i,j}}{{\tilde{x}}_{i}}$. *A*_leaf_, net leaf CO_2_ assimilation; *g_s_*, leaf stomatal conductance; iWUE_leaf_, leaf intrinsic water‐use efficiency; *A*_canopy,ground_, canopy CO_2_ assimilation ground flux; *A*_canopy,LA_, leaf area‐normalized canopy CO_2_ assimilation ground flux; *g_C_*, canopy conductance; iWUE_canopy_, canopy intrinsic water‐use efficiency; iWUE_biomass_, biomass‐based intrinsic water‐use efficiency

Among the different phenotypes measured, leaf and canopy gas exchange traits (*g_s_*, iWUE_leaf_, *A*_canopy,ground_), total LA, and leaf area exposure showed the greatest variation among the different cowpea lines (Figure 1). There was little variation among cowpea line in the number of nodes, stem angle, leaf length, and estimated chlorophyll content (SPAD). On average, the range of variability observed in other traits including stem length, canopy width, leaf and shoot mass was ± 50% relative to the median (Figure 1).

### 3.2 Correlations among canopy gas exchange traits

**Figure 2:**
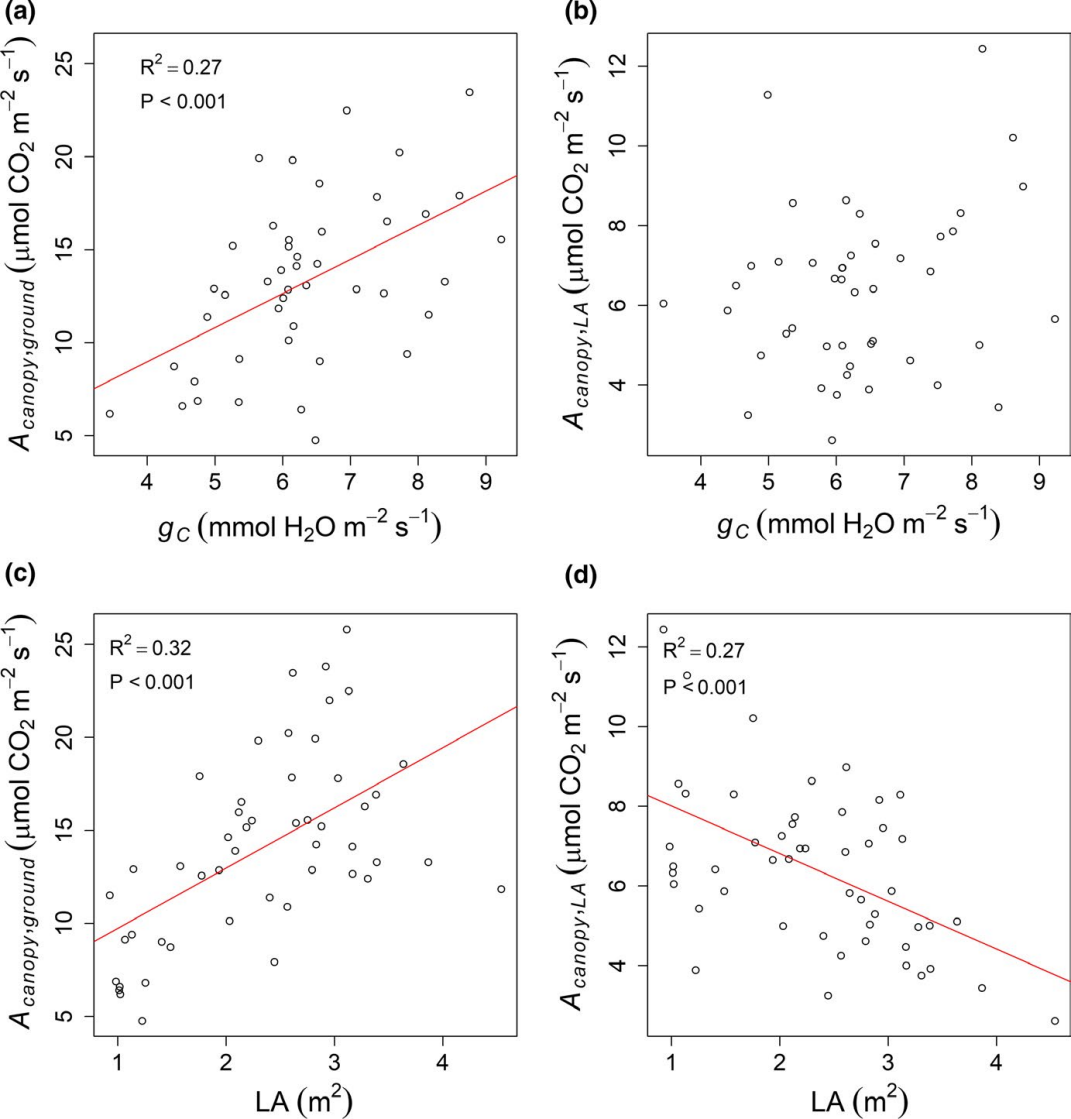
Linear regression between *A_canopy_* and canopy conductance (*g_C_*) and leaf area (LA)

There was a significant positive linear relationship between *A*_canopy,ground_ with canopy conductance, *g_C_* (*R* ^2^ = .27, *p*‐value < .001; Figure 2a). The correlation between *A* and *g_s_* at the leaf‐level was stronger than at the canopy‐level (Supporting information 1). As expected, greater total LA was also associated with greater *A*_canopy,ground_ (*R* ^2^ = .32, *p*‐value < .001; Figure 2c). When *A*_canopy_ was expressed on a leaf area basis, not ground area basis, there was a negative linear relationship between *A*_canopy,LA_ and total LA (*R* ^2^ = .27, *p*‐value < .001; Figure 2d), which may be indicative of increasing self‐shading in lines with greater leaf area.

**Figure 3:**
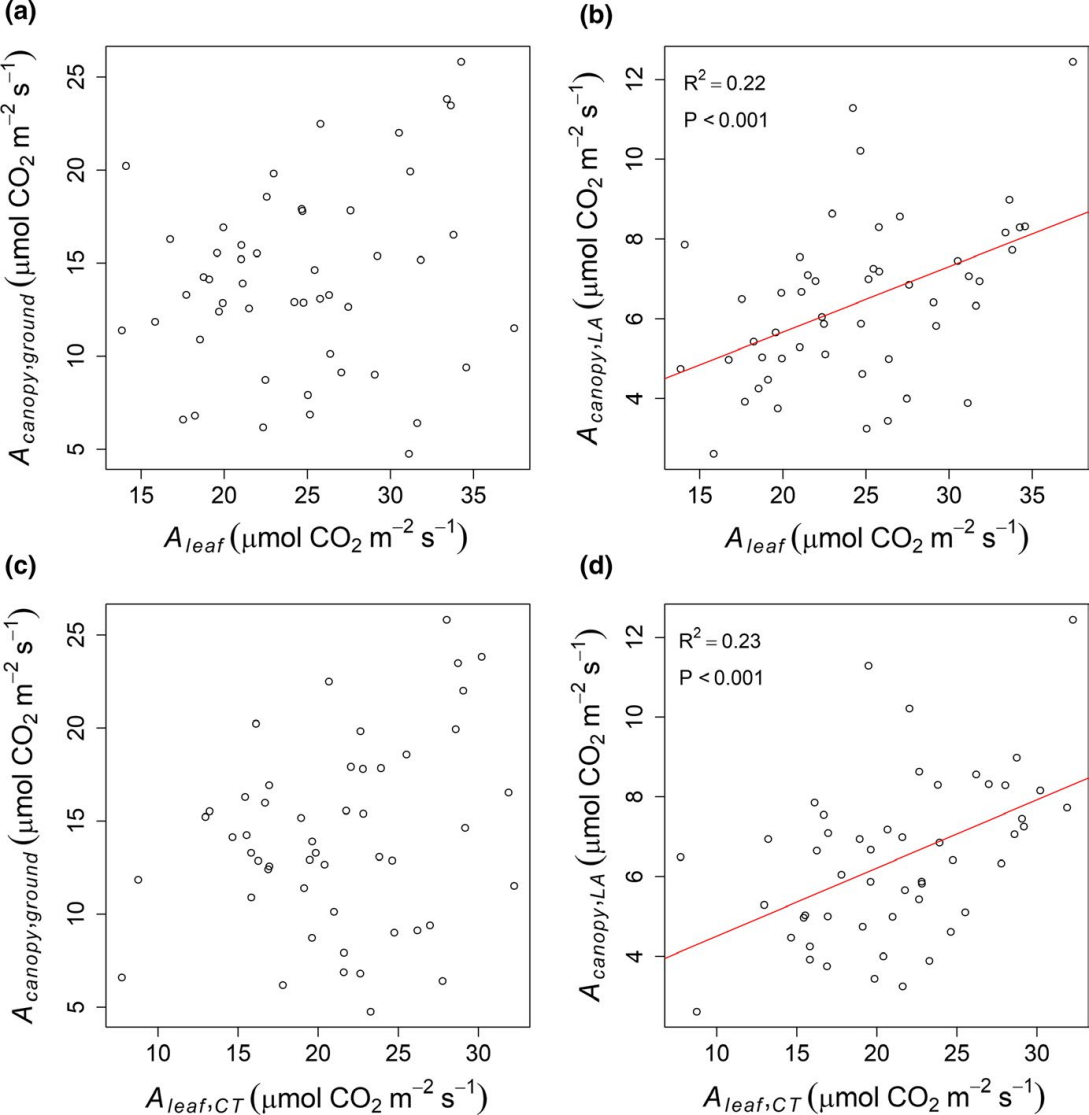
Linear regression between *A_canopy_* and leaf photosynthesis measured with a light source (*A*_leaf_) or with clear‐top chamber (*A*_leaf,CT_)

Leaf‐level photosynthetic measurements were not predictive of *A*_canopy,ground_ but exhibited a positive linear relationship with *A*_canopy,LA_ (Figure 3). Leaf photosynthesis measurements performed with a clear‐top chamber or a saturated‐light source performed equally at predicting A_canopy_ (Figure 3), probably because measurements were performed at noon when ambient light was saturating (i.e., ~1,500 μmol m^‐2^ s^‐1^). For this reason, only measurements of leaf photosynthesis performed with a light source were used in subsequent analysis.

### 3.3 General linear model to explain observed variability in canopy gas exchange

In a second analysis, the best minimum adequate model to predict both ground flux and LA‐normalized canopy CO_2_ assimilation was selected using a stepwise algorithm based on the AIC. This allows identification of the major predictors for *A*_canopy_ and traits with a considerable influence on canopy carbon assimilation. The best model predicting *A*_canopy,ground_ had a *R* ^2^ = .76 (*p*‐value < .001) while the best model predicting *A*_canopy,LA_ had a *R* ^2^ = .66 (*p*‐value < .001). Both models included leaf‐level photosynthesis, SPAD, leaf mass, canopy width, stem angle, and leaf area exposure.

**Figure 4:**
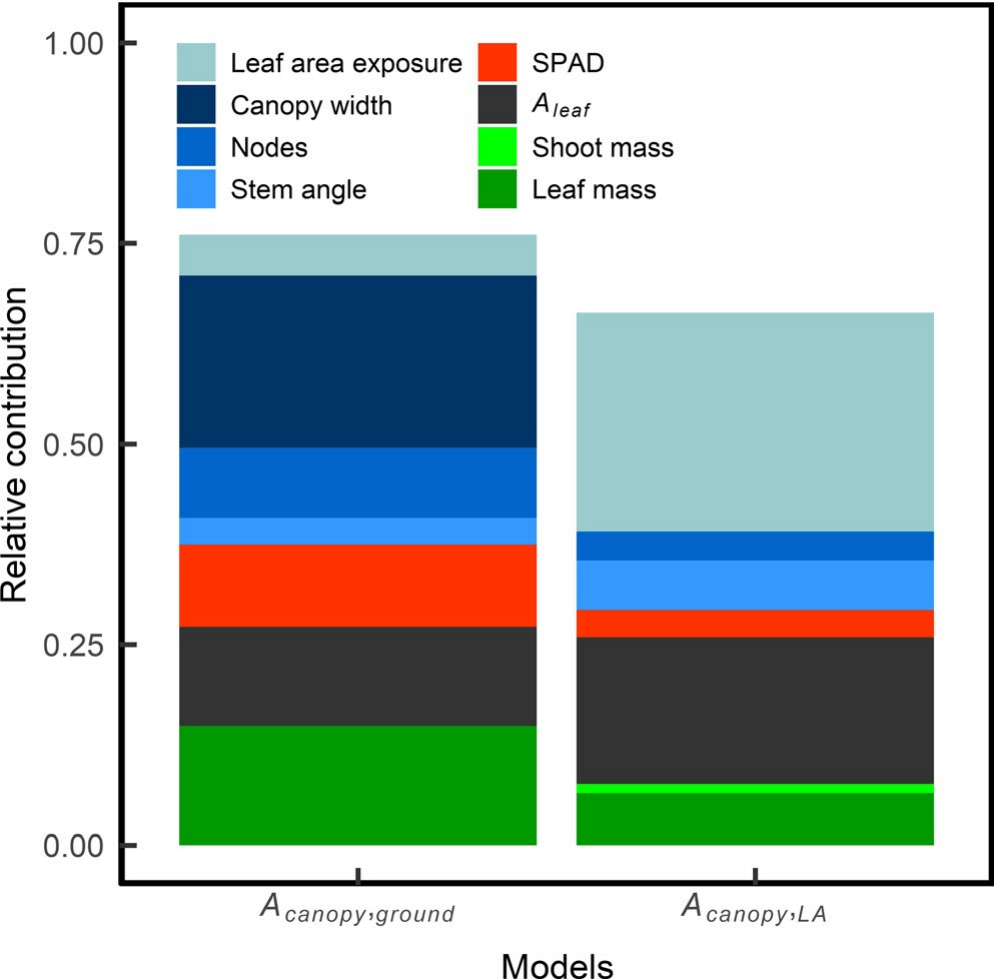
Relative contribution of the different parameters used in the minimum adequate models to explain the variation in (right) canopy CO_2_ assimilation ground flux and (left) leaf area‐normalized canopy CO_2_ assimilation ground flux. Parameters in green are related to the canopy biomass. Parameters in blue are related to the canopy architecture

The total variance explained by the models was then decomposed in order to evaluate the relative variance explained by each predictor variable (Figure 4). For both models, plant architecture (e.g., stem angle, number of nodes, canopy width and leaf area exposure) and *A*_leaf_ played comparable roles in the determination of canopy carbon assimilation. Traits related to the plant architecture explained 38.6 and 37.0% of the variance observed for *A*_canopy,ground_ and *A*_canopy,LA_, respectively. Leaf photosynthetic activity explained 12.3 and 18.2% of variance in *A*_canopy,ground_ and *A*_canopy,LA_, respectively. Biomass‐related traits had a more predominant role in *A*_canopy,ground_, by explaining 14.9% of total variance compared to 7.6% of variance in *A*_canopy,LA_. This indicates that canopy CO_2_ assimilation is not only determined by the amount of biomass and the *A*_leaf_ but also by traits defining the plant architecture.

### 3.4 Determination of groups with contrasted canopy architecture

**Figure 5:**
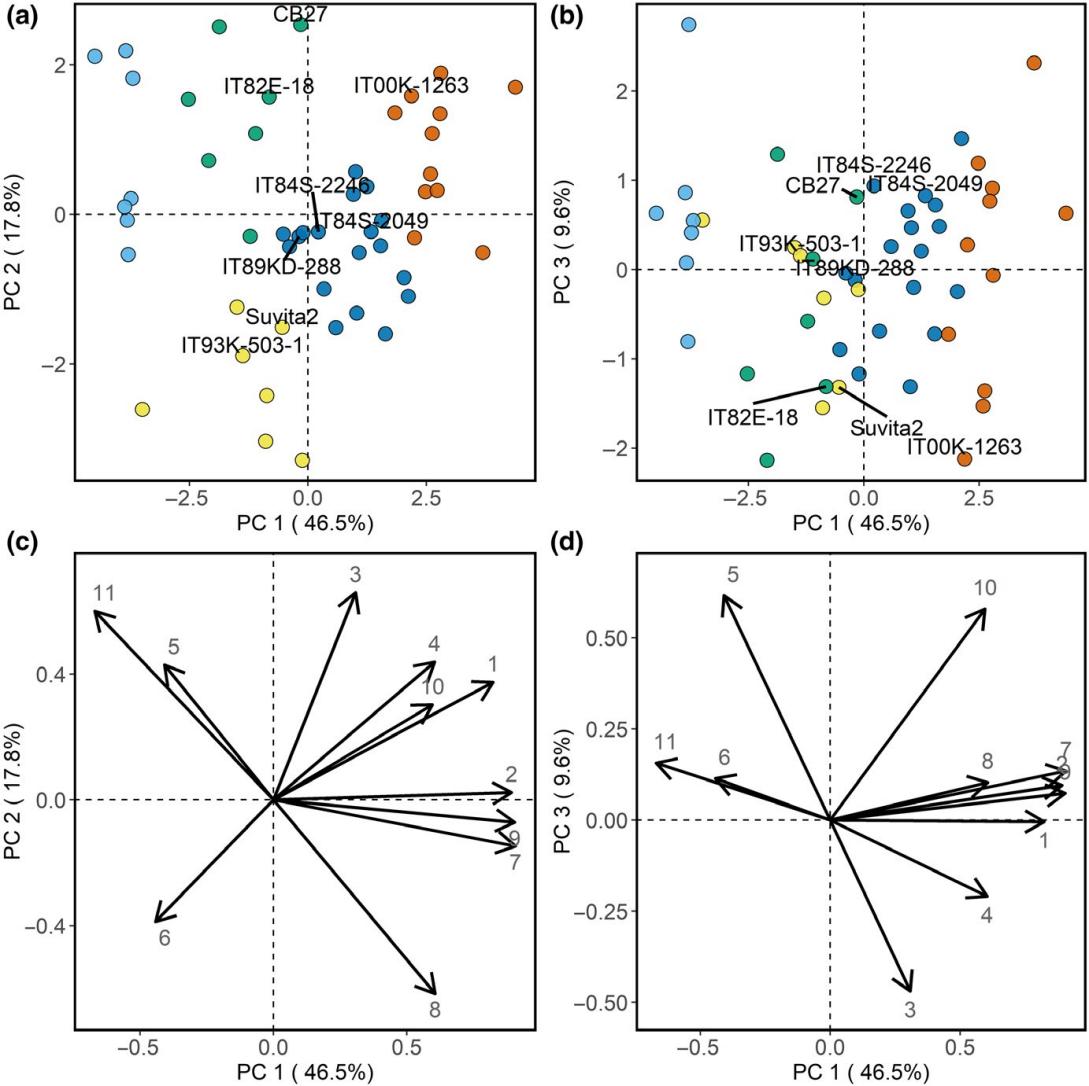
Clusters of similar and contrasted canopies. Determination of clusters with contrasted phenotypes was obtained by principal component analysis (PCA) followed by clustering. Distribution according to the principal components 1–2 (a) and 1–3 (b) and the correlation circles with the variables loaded on the principal components 1–2 (c) and 1–3 (d) are represented. The clusters have been highlighted by different colors with clusters 1, 2, 3, 4, and 5 in light blue, green, yellow, dark blue, and red. Canopy traits entered as variables in the PCA were as follows: 1—canopy width; 2—canopy height; 3—leaf length; 4—leaf width; 5—SPAD; 6—stem angle; 7—stem length; 8—nodes; 9—leaf mass; 10—shoot mass; 11—leaf area exposure

Five clusters with contrasting canopy architectures were defined by PCA‐HCPC (Figure 5). The average trait values for the defined clusters are given in Table 1. The first principal component (PC1) explained 46.5% of the variance observed, with stem length, total leaf mass, canopy height, and canopy width as the main contributors. The clusters were mainly separated on the PC1. PC2 was mostly determined by leaf length, the number of nodes, and leaf area exposure. Leaf length was also an important contributor to PC3, along with SPAD and the shoot mass. PC3, however, did not allow clusters to segregate. Clusters 1 and 2 were located in the third quadrants characterized by high LA, SPAD value, and a low node number (Figure 5). Cowpea lines located in cluster 1 showed a phenotype that strongly differed from the parents and was characterized by significantly lower leaf mass (*p*‐value < .001, Table 1).

**Table 1:** Average (+‐ *SD*) in the different clusters defined in Figure 5

Traits	Cluster 1 (*n* = 7)	Cluster 2 (*n* = 7)	Cluster 3 (*n* = 7)	Cluster 4 (*n* = 18)	Cluster 5 (*n* = 11)	*p*‐values
*A*_leaf_	27.95 ± 7.02 a	23.81 ± 3.47 a	22.83 ± 4.43 a	25.38 ± 6.49 a	20.75 ± 4.64 a	ns
*g_s_*	0.76 ± 0.45 a	0.42 ± 0.18 a	0.39 ± 0.31 a	0.48 ± 0.29 a	0.29 ± 0.17 a	ns
iWUE_leaf_	45.67 ± 19.14 a	66.05 ± 30.59 a	75.37 ± 26.39 a	65.84 ± 25.93 a	87.64 ± 30.61 a	ns
*A*_canopy,ground_	8.01 ± 2.03 c	11.1 ± 2.9 bc	12.27 ±4.72 abc	16.34 ± 4.36 a	15.88 ± 3.65 ab	***
*A*_canopy,LA_	7.88 ± 2.24 a	7.38 ± 1.97 ab	5.92 ± 2.44 ab	6.45 ± 1.59 ab	4.84 ± 1.62 b	*
*g_c_*	5.76±1.75 a	5.57 ± 0.81 a	6.83 ± 1.56 a	6.86 ± 2.13 a	6.92± 0.95 a	ns
iWUE_canopy_	1.43 ± 0.27 c	2.01 ± 0.52 abc	1.79 ± 0.54 bc	2.43 ± 0.5 a	2.32 ± 0.54 ab	***
iWUE_biomass_	45.49 ± 18.67 a	48.4 ± 7.44 a	38.64 ± 11.75 a	46.87 ± 11.78 a	52.73 ± 10.02 a	ns
SPAD	73.71 ± 5.95 a	68.3 ± 6.01 ab	60.41 ± 7.46 b	64.64 ± 4.93 b	63.51 ± 7.17 b	**
Leaf length	8.77 ± 1.95 ab	9.96 ± 0.48 a	7.64 ± 0.86 b	9.47 ± 0.52 a	10.12 ± 1.15 a	***
Leaf width	4.17± 0.71 b	5.8 ± 0.7 a	4.41 ± 0.35 b	5.33 ± 0.7 a	5.92 ± 0.62 a	***
Leaf area	1.02 ± 0.06 d	1.52± 0.3 cd	2.11 ± 0.53 bc	2.56 ± 0.41 b	3.4 ± 0.52 a	***
Leaf area exposure	42.61 ± 3.46 a	38.8 ± 8.21 a	22.94 ± 6.82 b	25.46 ± 3.51 b	24.78 ± 4.36 b	***
Stem angle	78.46± 1.87 a	75.82 ± 4.18 a	81.39 ± 5.66 a	75.53 ± 11.55 a	57.44 ± 16.07 b	***
Stem length	27.07 ± 3.28 d	38.32± 7.81 cd	50.11 ± 10.39 c	62.92 ± 10.24 b	76± 14.04 a	***
Nodes	13.04 ±1.93 b	14.75 ±1.94 b	18.39 ± 1.27 a	18.38± 1.68 a	17.52 ± 1.98 a	***
Canopy width	43.43 ± 3.36 d	57.49 ± 8.35 bc	46.51 ± 11.16 cd	64.35 ± 8.44 b	82.74 ± 8.08 a	***
Canopy height	33.76 ± 4.46 c	42.7 ± 7.51 b	41.26± 4.82 bc	50.91 ± 5.25 a	56.2 ± 4.56 a	***
Leaf mass	94 ± 12.21 d	124.43± 13.43 c	129.86 ± 20.92 c	150.8 ± 19.6 b	175.7 ± 18.1 a	***
Shoot mass	141.71 ± 20 bc	142.71 ± 38.65 bc	119.71 ± 16.11 c	153.33 ± 22.28 ab	182.3 ± 31.3 a	***
Total biomass	235.71 ± 22.63 c	267.14 ±42.94 bc	249.57 ± 25.57 c	304.13 ± 38.65 b	358 ± 43.99 a	***

Note

Statistical differences among clusters are assessed by *p*‐values from the linear models where ns, *, **, and *** indicate *p*‐values > .05, <.05, <.01, and < .001. Different letters indicate significant differences among the clusters (Tukey's HSD, ɑ = 0.05). In the Figures, the clusters are represented by different colors with clusters 1, 2, 3, 4, and 5 shown in light blue, green, yellow, dark blue, and red.

**Figure 6:**
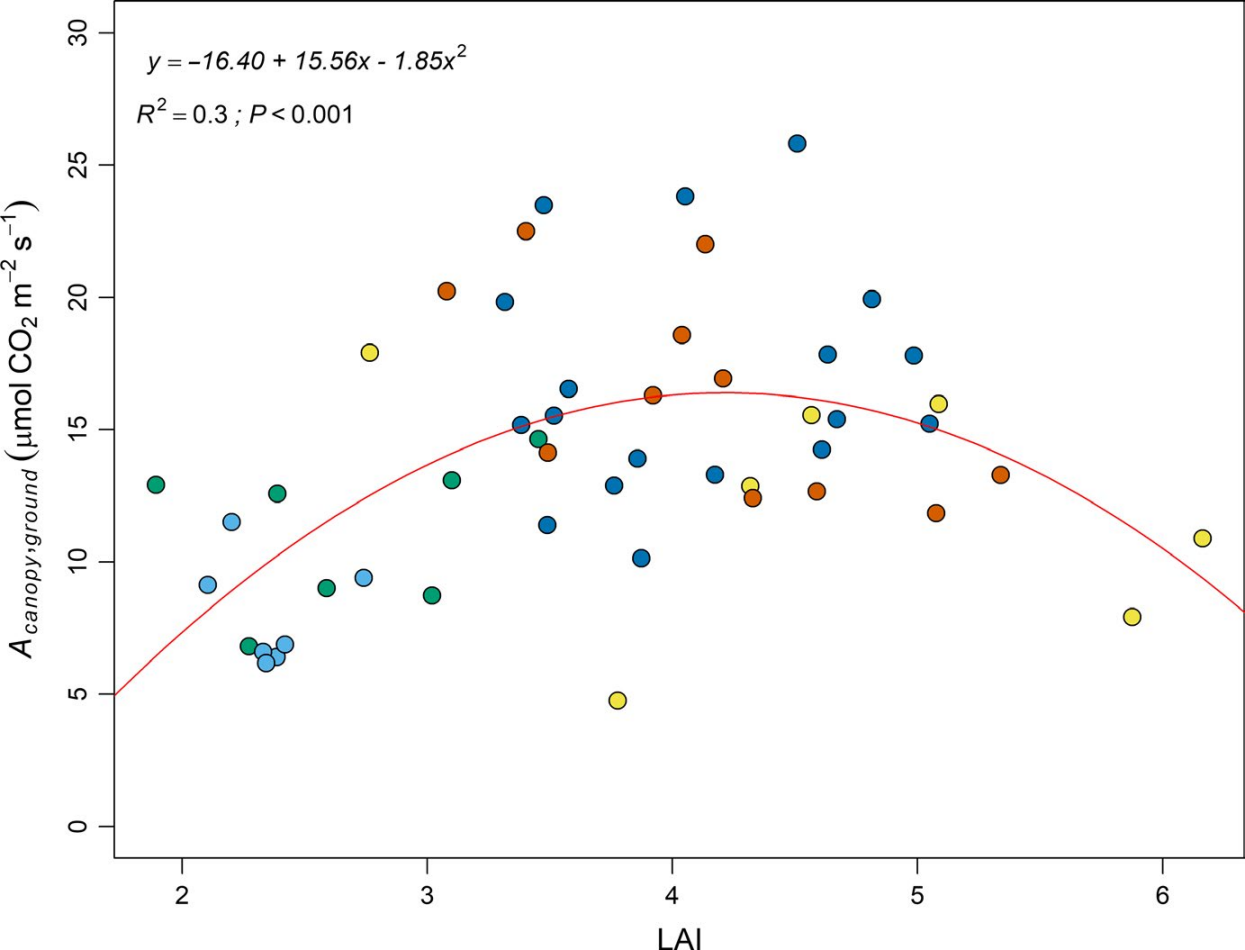
Relationship between *A*_canopy,ground_ and leaf area index (LAI). The best fit was obtained using a second order polynomial regression (red line). This model was used to estimate the LAI value for which *A*_canopy,ground_ was maximum. The points are colored based on the different clusters previously defined. The same color code was been used, with clusters 1, 2, 3, 4, and 5 shown in light blue, green, yellow, dark blue, and red

Clusters 3, 4, and 5 did not differ significantly in leaf and canopy photosynthesis despite showing significant differences in biomass (i.e., shoot, leaf and total biomass) (Table 1). Increasing LA in those clusters did not translate into an increase in canopy photosynthesis, which suggests differences in the efficiency of light interception with different canopy architectures. This is supported by the non‐linear relationship between *A*_canopy,ground_ and LAI (Figure 6). Leaf area exposure was also not significantly different among clusters 3, 4 and 5.

### 3.5 *Relationship between A*_canopy,ground_ *and A*_canopy,LA_ *under different canopy architecture*

**Figure 7:**
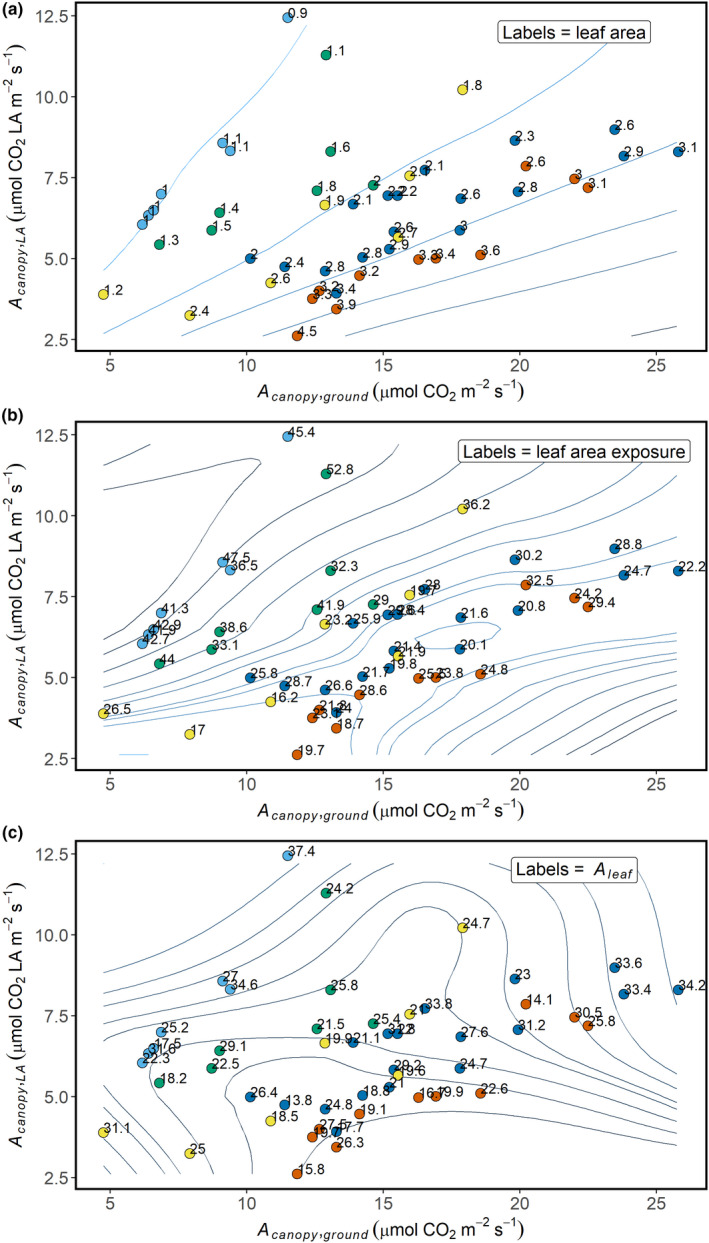
Relationship between *A*_canopy,ground_ and *A*_canopy,LA_ with different canopy traits. Labels show (a) leaf area, (b) leaf area exposure represented by $\frac{\text{canopy width}}{\text{leaf area}}$ and (c) leaf photosynthesis. Isoline values (blue lines) provide visual support for the distribution of the different parameters and were produced by local polynomial regression for $\text{Trait}=f\left(A_{\text{canopy,ground}},A_{\text{canopy,LA}}\right)$ using the Loess R function. The points are colored based on the different clusters previously defined. The same color code was been used, with clusters 1, 2, 3, 4, and 5 shown in light blue, green, yellow, dark blue, and red

The correlation between *A*_canopy,ground_ and *A*_canopy,LA,_ differed among clusters with contrasting canopy architecture (Figure 7). Comparison of the slopes from the linear regression revealed a significant difference between cluster 1 and clusters 3, 4, and 5. This may be explained by the very low LA for lines in cluster 1 that led to a strong relationship between *A*_canopy,ground_ and *A*_canopy,LA_ (*R* ^2^ = .93, *p*‐value < .001). This relationship was weaker in the other clusters, except in cluster 5 with very high leaf area canopies (*R* ^2^ = .89, *p*‐value < .001).

The linear regressions between canopy traits with either *A*_canopy,ground_ or *A*_canopy,LA_ were explored within each cluster to assess if traits determining canopy CO_2_ assimilation varied with differences in canopy architecture. Because of the similarities between the clusters 1 and 2 and the small size of the two clusters, they were pooled for this analysis. Most of the traits explaining observed variation in A_canopy_ were significant for only one cluster (Table 2), which could be explained by low variation of traits within each cluster. Increase in LA strongly contributed to increase in *A*_canopy,ground_ (*R* ^2^ = .45, *p*‐value < .01) in clusters 1 and 2 as expected for low leaf area cowpea lines. Total LA, however, showed a negative relationship with *A*_canopy,LA_ (*R* ^2^ = .61, *p*‐value < .01) in cluster 5, which was characterized by high biomass. SPAD values were also negatively correlated with *A*_canopy,ground_ (*R* ^2^ = .51, *p*‐value < .05) and *A*_canopy,LA_ (*R* ^2^ = .49, *p*‐value < .05) in cluster 5, which may suggest that light green leaves contributed to improved light diffusion within dense canopies. Leaf area exposure significantly contributed to the variation in *A*_canopy,ground_ (*R* ^2^ = .42, *p*‐value < .05) and *A*_canopy,LA_ (*R* ^2^ = .60, *p*‐value < .01) in cluster 5. This emphasizes the importance of light distribution for canopy CO_2_ assimilation in dense cowpea canopies.

**Table 2:** Linear regression of A_canopy_ with other traits within each cluster

Clusters	Traits	*A*_canopy,ground_			*A_canopy,LA_*		
		Slopes	*R* ^2^	*p*‐values	Slopes	*R* ^2^	*p*‐values
1&2 (*n* = 14)	*A*_canopy,ground_	‐	‐	‐	0.39	.31	*
	*A*_canopy,LA_	0.79	.31	*	‐	‐	‐
	iWUE_canopy_	4.29	.54	**	0.98	.06	ns
	*A*_leaf_	0.1	.04	ns	0.2	.32	*
	Leaf area	5.81	.45	**	−1.43	.05	ns
	Canopy width	0.18	.35	*	0	0	ns
1 (*n* = 7)	*A*_canopy,ground_	‐	‐	‐	1.06	.93	***
	*A*_canopy,LA_	0.87	.93	***	‐	‐	‐
	*g_c_*	0.93	.65	*	0.96	.56	ns
	Leaf mass	−0.14	.67	*	−0.15	.72	*
2 (*n* = 7)	*A*_canopy,ground_	‐	‐	‐	0.44	.42	ns
	*A*_canopy,LA_	0.95	.43	ns	‐	‐	‐
	iWUE_canopy_	4.75	.71	*	2.66	.49	ns
3 (*n* = 7)	*A*_canopy,ground_	‐	‐	‐	0.44	.73	*
	*A*_canopy,LA_	1.65	.73	*	‐	‐	‐
	iWUE_canopy_	7.06	.65	*	2.89	.40	ns
	Canopy width	0.34	.65	*	0.15	.45	ns
4 (*n* = 18)	*A*_canopy,ground_	‐	‐	‐	0.29	.69	***
	*A*_canopy,LA_	2.37	.69	***	‐	‐	‐
	*g_c_*	1.51	.54	**	0.38	.26	*
	*A*_leaf_	0.47	.46	**	0.17	.49	**
	*g_s_*	11.05	.64	***	3.34	.48	**
	iWUE_leaf_	−0.12	.48	**	−0.03	.31	*
	Leaf length	−3.49	.22	*	−0.65	.06	ns
	Stem angle	0.13	.11	ns	0.08	.30	*
	Nodes	1.37	.27	*	0.38	.17	ns
	Shoot mass	0.11	.30	*	0.02	.08	ns
	Total biomass	0.06	.26	*	0.01	.04	ns
5 (*n* = 11)	*A*_canopy,ground_	‐	‐	‐	0.42	.89	***
	*A*_canopy,LA_	2.14	.89	***	‐	‐	‐
	iWUE_canopy_	5.78	.72	**	2.25	.55	*
	SPAD	−0.41	.51	*	−0.18	.49	*
	Leaf area	−4.40	.33	ns	−2.63	.61	**
	Leaf area exposure	0.61	.42	*	0.32	.60	**

Note

Only traits with a *p*‐value < .05 for the linear regression are shown. ns, *, **, and *** indicate a > 0.05, <0.05, <0.01, and < 0.001 *p*‐value for the linear regression. Additional reported values are slope and coefficient of determination (*R* ^2^). In the Figures, the clusters are represented by different colors with clusters 1, 2, 3, 4, and 5 shown in light blue, green, yellow, dark blue, and red.

### 3.6 Linear regression of intrinsic water‐use efficiency with canopy traits

**Figure 8:**
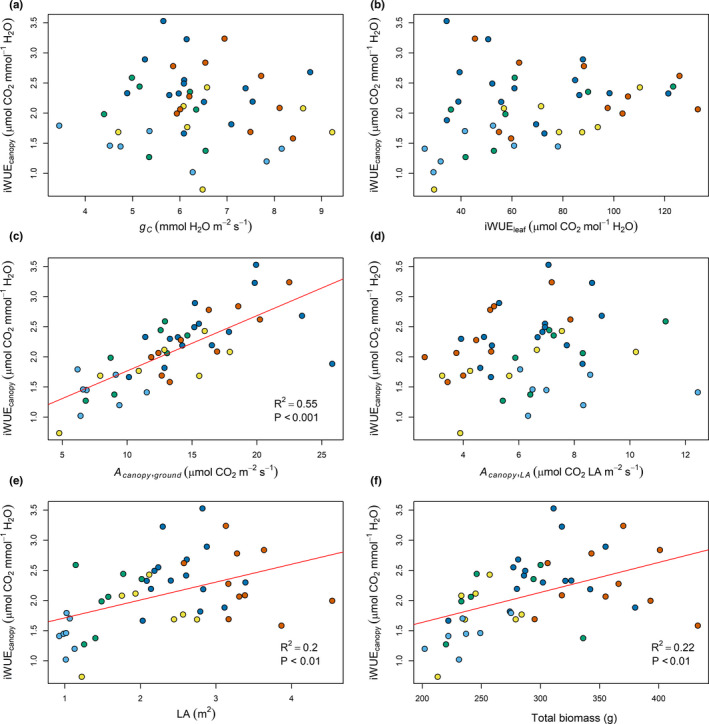
Linear regression of canopy intrinsic water‐use efficiency (iWUE_canopy_) with varied traits. Genotypes are colored based on clustering (Figure 5) with clusters 1, 2, 3, 4, and 5 shown in light blue, green, yellow, dark blue, and red

**Figure 9:**
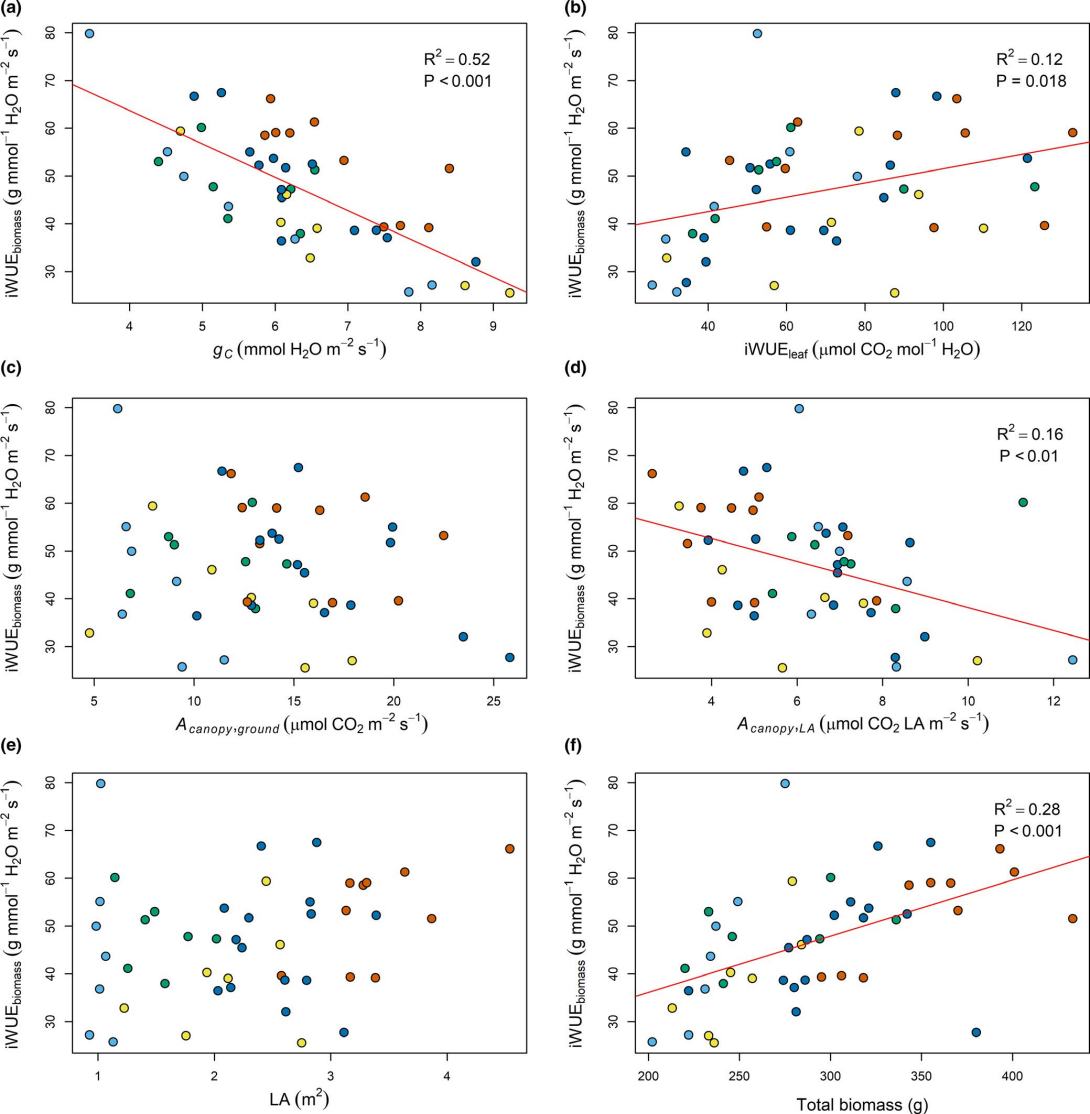
Linear regression of canopy biomass‐based intrinsic water‐use efficiency (iWUE_biomass_) with varied traits. Genotypes are colored based on clustering (Figure 5) with clusters 1, 2, 3, 4, and 5 shown in light blue, green, yellow, dark blue, and red

Variability in iWUE_canopy_ could not be explained by canopy conductance (Figure 8). However, iWUE_canopy_ showed a positive linear relationship with canopy CO_2_ assimilation (*R* ^2^ = .55, *p*‐value < .001). Genotypes from clusters 4 and 5, characterized by the highest *A*_canopy,ground_, also showed the highest iWUE_canopy_ values. Positive linear relationships between iWUE_canopy_ and total leaf area and biomass (*R* ^2^ = .20, *p*‐value < .01 and *R* ^2^ = .22, *p*‐value < .01, respectively) were also observed and may be explained by the positive contribution these traits can have on canopy CO_2_ assimilation. In contrast with iWUE_canopy_, iWUE_biomass_ was negatively correlated to canopy conductance (*R* ^2^ = .52, *p*‐value < .001; Figure 9a). iWUE_canopy_ also showed a positive linear relationship with total biomass (*R* ^2^ = .28, *p*‐value < .001; Figure 9f). Interestingly, iWUE_canopy_ only explained 10.7% (*p*‐value < .05) of iWUE_biomass_ variance suggesting that the ability to produce more biomass per unit of water was not explained by a better efficiency to assimilate CO_2_.

### 3.7 General linear model to explain observed variability in intrinsic water‐use efficiency

The best minimum adequate model to predict iWUE_canopy_ both on a CO_2_ assimilation and biomass basis was selected using a stepwise algorithm based on the AIC. Only traits related to the canopy architecture and *A*_leaf_ were added to the model in order to assess their contribution to the variance observed iWUE_canopy_. The best model for the prediction of iWUE_canopy_ had a *R* ^2^ = .56 (*p*‐value < .001) while the best model predicting iWUE_biomass_ had a *R* ^2^ = .64 (*p*‐value < .001). Models only shared canopy width in common.

**Figure 10:**
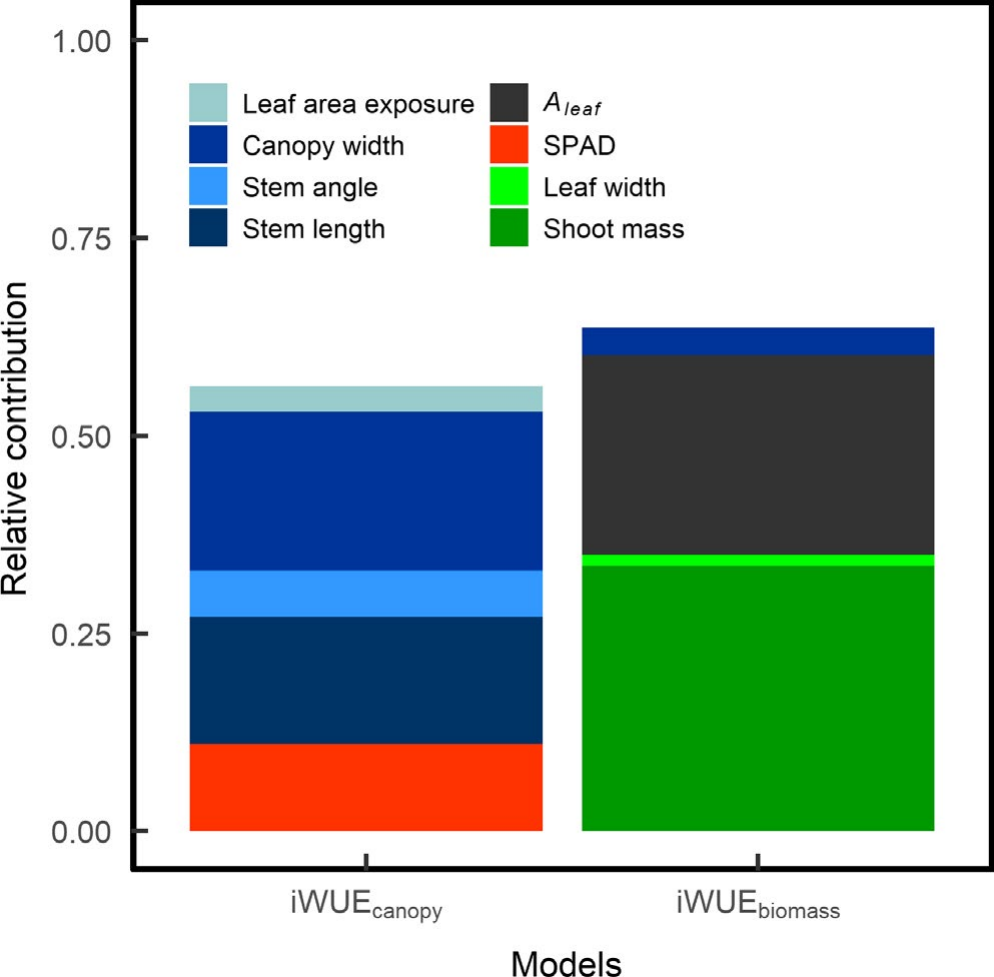
Relative contribution of the different parameters used in the minimum adequate models to explain the variation in (left) canopy intrinsic water‐use efficiency and (right) biomass‐based intrinsic water‐use efficiency. Parameters in green are related to the canopy biomass. Parameters in blue are related to the canopy architecture

The partitioning of the relative contribution of each predictor variable in the models revealed that the two models had different drivers. About 45% of the variation observed in iWUE_canopy_ was explained by canopy architectural traits including stem angle, stem length, canopy width, and leaf area exposure (Figure 10). In contrast, the model for iWUE_biomass_ was mainly determined by plant biomass (33.5%). *A*_leaf_ also had strong predictive power for iWUE_biomass_ by explaining 25.3% of the variance observed but was not selected in the linear model for iWUE_canopy_. However, most of the traits included in the iWUE_canopy_ model were also included in the A_canopy_ model.

## 4 DISCUSSION

### 4.1 Drivers of canopy CO_2_ assimilation

Significant variability was observed in canopy CO_2_ assimilation (4.8–25.8 μmol CO_2_ m^‐2^ s^‐1^) among the 50 cowpea genotypes. This variation could be partly explained by leaf mass (14.9%), *A*_leaf_ (12.3%), chlorophyll content (SPAD value; 10.2%) and canopy architecture (38.6%). Our study highlights the diversity in canopy traits among a cowpea MAGIC population and emphasizes the multifactorial nature of canopy photosynthesis. The importance of canopy architecture for canopy photosynthesis has been highlighted in soybean (Song, Srinivasan, Long, & Zhu, 2019). Using a 3D canopy model and ray‐tracing, the canopy structure was estimated to account for 4.8%–20% of the variation in canopy photosynthesis depending on the growth stage and ambient light conditions (Song et al., 2019). The greater contribution of canopy architecture to canopy photosynthesis observed in our study could be explained by the variety of phenotypes used in our study and the uniformity of environmental conditions when estimated canopy photosynthesis, while Song et al. (2019) modeled canopy CO_2_ through the entire growing season, but for a single soybean cultivar.

### 4.2 Efficiency of canopy photosynthesis

Genotypes with canopies characterized by a high leaf area and/or covering more ground area expressed the highest values for *A*_canopy,ground_ as they were able to intercept more incident radiation (Table 1, Figure 2, Supporting information 1). Our analysis suggests that light penetration was a limiting factor in canopy photosynthesis, especially for genotypes with high biomass. This finding supports other studies that showed an advantage of erect leaves on canopy carbon assimilation, especially in canopies with high LAI, because erect leaves allowed greater light penetration (Loomis & Williams, 1969; Pepper, Pearce, & Mock, 1977; Sinclair & Muchow, 1999). Thus, traits favoring the exposure of greater leaf area to irradiance (i.e., a wider canopy relative to its total leaf area and lighter green leaves) may contribute to improving overall canopy CO_2_ assimilation. Greater canopy photosynthetic activity with improved light distribution in canopies has been previously reported by several studies (Burgess, Retkute, Herman, & Murchie, 2017; Li et al., 2014; Song, Zhang, & Zhu, 2013), but has not been studied in cowpea. Better light penetration within the canopy may also contribute to delayed senescence of leaves located in the lowest layer of the canopy (Liebsch & Keech, 2016) contributing to the maintenance of leaf area later during the season eventually leading to higher yield (Koester, Skoneczka, Cary, Diers, & Ainsworth, 2014; Liu et al., 2015).

We found that canopies with significantly lower LA (e.g., cluster 4 versus cluster 5; Table 1) had similar canopy CO_2_ assimilation to canopies with greater leaf area, probably because the canopy leaf area was more efficiently used (Table 1, Figure 7) with less self‐shading. Lower leaf area canopies may also have lower respiratory activity compared to higher biomass canopies, as maintenance respiration cost is linearly dependent on plant biomass (Amthor, 2000; Ryan, 1991). Similar results were found in situ for soybean where a 4% reduction in leaf area by excision did not lead to a decline in CO_2_ assimilation, presumably due to a reduction in canopy respiration and self‐shading (Srinivasan et al., 2016). In maize, the removal of two leaves above the ear leaf led to an increase of canopy photosynthesis up to 14% (Liu et al., 2015). Both studies saw an increase in seed yield in response to the reduction in leaf area. The lowest leaf area index (LAI) for which the maximum net primary productivity and yield were achieved in soybean was 4.0 (Srinivasan et al., 2016), while in maize reported LAI values for maximum yield ranged from 2.6 to 6.9 at maturity (Liu et al., 2017). In our study, cowpea CO_2_ assimilation started to plateau in cluster 4 when an increase in LA did not result in greater *A*_canopy,ground_. The optimum LAI value for cowpea for net canopy photosynthesis in our study was 4.19 (Figure 6). Additional studies are needed assess if this range also translates into maximum bean yield.

Light green leaves were associated with greater canopy photosynthesis (Supporting information 2), and leaf greenness explained about 50% of the variation in *A*_canopy,ground_ and *A*_canopy,LA_ in genotypes in cluster 5, which were characterized by high LA (Table 2). Using published relationships between SPAD values and chlorophyll content for coffee plants (Torres Netto, Campostrini, De Oliveira, & Bressan‐Smith, 2005), citrus cultivars (Jifon, Syvertsen, & Whaley, 2005), soybeans (Markwell, Osterman, & Mitchell, 1995), *Castanopsis carlessi* (Wang et al., 2009), *Spathiphyllum* Schott (Wang, Chen, & Li, 2004), and cowpeas (Murillo‐Amador et al., 2004), the reduction in leaf greenness associated with the increase in canopy photosynthesis observed in the cluster 5 could have represented a 30 ± 7% reduction in leaf chlorophyll content (μmol/m^2^). The resulting enhanced canopy photosynthesis could be explained by increased transmission of light to the lower canopy, due to a reduced leaf absorbance. This hypothesis is supported by a previous modeling study in rice that predicted a moderate increase in canopy photosynthesis in response to a reduction in canopy chlorophyll content (Song, Wang, Qu, Ort, & Zhu, 2017). A modeling study with soybean, however, did not show an increase in canopy photosynthesis in response to a reduction in chlorophyll content (Walker et al., 2017). Instead, canopy photosynthesis remained constant after a ~ 50% reduction in chlorophyll content (depending on the assumptions made by the model), and only after greater reductions in chlorophyll was photosynthesis reduced. The decline in canopy CO_2_ assimilation was explained by an increase in leaf reflectance leading to a reduction in light absorbance by the canopy. This contrasting result may be caused by different reasons. One possible explanation could be the 100–500 μmol/m^2^ range of chlorophyll content assumed in soybean study (Walker et al., 2017). Based on the relationship performed by Murillo‐Amador et al. (2004) in 60 cowpea cultivars, the range of chlorophyll content observed in the cluster 5 is estimated at 537–656 μmol/m^2^_,_ which is within the range of values commonly reported for cowpea (Jemo et al., 2017; Singh & Raja Reddy, 2011). The soybean study also assumed that chlorophyll declined from the top to the bottom of the canopy (Drewry et al., 2010; Walker et al., 2017), which may not be representative of field observations (Ciganda, Gitelson, & Schepers, 2008; Kong et al., 2017; Winterhalter, Mistele, & Schmidhalter, 2012). Our results complement previous studies by emphasizing that, in canopies of high leaf area and high chlorophyll content, canopy photosynthesis can benefit from a reduction in chlorophyll content. Further studies are needed to clarify the range of reduction in chlorophyll that can lead to an increase in canopy photosynthesis as well as how leaf area can affect the canopy response.

### 4.3 Cowpea water‐use efficiency

A cowpea MAGIC population was developed by inter‐crossing lines that were able to produce high yields under drought conditions (Huynh et al., 2018). While there was no significant linear relationship between iWUE_canopy_ and *g_c_*, we found a strong positive linear relationship between iWUE_canopy_ and A_canopy_ (Figure 8). Different adaptation mechanisms may exist in different cowpea lines in response to drought events. Drought avoidance responses include developing a more efficient root system to maximize water uptake (Munjonji, Ayisi, Boeckx, & Haesaert, 2018) or reducing the leaf area and transpiration surface, which contributes to lower water demand by the canopy (Anyia & Herzog, 2004; Bastos, Nascimento, Silva, Freire Filho, & Gomide, 2011). These strategies allow maintenance of high leaf *g_s_* and transpirational cooling under drought (Munjonji et al., 2018). Drought tolerance in cowpeas can also be achieved by reduction of stomatal conductance, allowing maintenance of a high relative water content in leaves (Anyia & Herzog, 2004; Bertolli, Rapchan, & Souza, 2012). The MAGIC population studied here was developed to assess genes involved in drought response in high‐yielding cultivars and consists of wide range of strategies to mitigate against drought stress, including early flowering (Huynh et al., 2018). The latter strategy enables completion of the reproductive cycle before the occurrence of a late drought event. This strategy was prevalent in cowpea lines with low biomass and low node number from clusters 1 and 2 where 100% and 43% of the lines were forming pods at the time of measurement. In contrast, only 14% and 28% of the lines in clusters 3 and 4, and no line in cluster 5 were forming pods at the time of measurement. This shows that early developing lines tended to have lower biomass, and that development may be associated with canopy architecture and photosynthesis. Even though there was no evidence of drought in this field experiment, the strong linear relationship observed between iWUE_canopy_ and A_canopy_ (Figure 8) also suggests that the water‐use efficiency of the population is partly explained by its ability to maintain high canopy photosynthesis without proportionally changing canopy conductance. This does not exclude a more predominant role of stomata in iWUE_canopy_ under drought conditions. While leaf iWUE is mainly determined by the interaction between leaf physiology and the environment, plant iWUE is determined by the combination of architectural and physiological characteristics of the crop along with energy exchange at the soil surface (Hatfield & Dold, 2019). Spreading growth habit has been described as a drought‐tolerant strategy and contributed to enhanced iWUE by providing better ground coverage, reducing the amount of radiation intercepted by the soil surface and thus, reducing soil evaporation (Sennhenn, Njarui, Maass, & Whitbread, 2017). We also found a positive correlation between the width of the canopy and iWUE_canopy_ (Pearson's correlation coefficient = 0.56, *p*‐value < .001; Supporting information 2), although this was found in the absence of drought stress.

### 4.4 Breeding strategies

Kamara et al. (2017) reported that increased cowpea total biomass contributed to greater seed yield, based on the positive correlations between canopy height and fodder yield with seed yield. Positive correlations between canopy height and plant biomass with canopy CO_2_ assimilation were found in our study (Pearson's correlation coefficient = 0.48, *p*‐value < .001; Supporting information 2), which may indicate that improvement in canopy photosynthesis may contribute to increased seed yield. Improvement of photosynthesis has been widely proposed as a key target for increasing crop yield (Simkin et al., 2019; Weber & Bar‐Even, 2019; Wu et al., 2019). Evidence of the benefit of increased photosynthetic activity for seed yield is supported by experiments in elevated CO_2_ for diverse crops (Ainsworth & Long, 2005) and legumes such as soybean (Morgan, Bollero, Nelson, Dohleman, & Long, 2005; Sanz‐Sáez et al., 2017). More studies are needed to determine the effectiveness of breeding for high A_canopy_ and its translation to high yield.

Potential yield increases as a result of increased canopy photosynthesis may however be mitigated by drought events in rain‐fed agricultural production systems, which is a common practice in sub‐Saharan Africa (Dingkuhn, Singh, Clerget, Chantereau, & Sultan, 2006; FAO, 2006; van Ittersum et al., 2016). Indeed, it was pointed that the decline in photosynthetic activity mediated by stomatal closure in response to drought events could be one of the factors responsible for the yield reduction in some cultivars (Munjonji et al., 2018; Rivas et al., 2016; Singh & Raja Reddy, 2011). Therefore, traits for improved CO_2_ canopy assimilation must be selected jointly with traits for high canopy water‐use efficiency. Breeding programs should also favor drought responses that allow the maintenance of high canopy CO_2_ assimilation in order to maximize the benefit of high canopy photosynthesis for seed yield. This is especially crucial as selections for lines with high CO_2_ canopy assimilation will also select for lines with higher canopy transpiration (Figure 2a). However, breeding for both drought tolerance and high photosynthetic capacity may be challenging, as Rivas et al. (2016) have observed a trade‐off between those two traits. This trade‐off may however be partly lifted by introducing more water‐use efficient canopy architecture such as phenotypes with a better ground coverage (Sennhenn et al., 2017), which would also benefit the overall canopy CO_2_ assimilation by exposing more leaves to solar radiation.

Traits such as canopy width, leaf mass, stem length, and canopy height were more strongly correlated with canopy photosynthesis than leaf‐level measurements of photosynthesis (Supporting information 2) and may represent better proxies for selection of lines with high canopy photosynthetic capacity. Those traits also showed a stronger correlation coefficient with *A*_canopy,ground_ (>0.7, Supporting information 2) than the total biomass (0.48, Supporting information 2). Total biomass alone only explained 28% of the variance observed in *A*_canopy,ground_ (*p*‐value < .001, Supporting information 7) compared to the 75% explained when several traits were used as predictor in the GLM (Figure 4). This may partly result from limited light penetration in high biomass canopies from the cluster 5. As shown in this study, canopy photosynthesis is a complex trait and is predicted by a combination of traits, including leaf photosynthesis. Targeting phenotypes with a LAI close to 4.2 at the reproductive stage may also allow the selection of canopies with maximum CO_2_ assimilation without overinvesting in leaf development or vegetative biomass, which may lessen canopy respiration, transpiration and improve light penetration, thus improving yield (Liu et al., 2017; Srinivasan et al., 2016).

Interestingly, iWUE_canopy_ and iWUE_leaf_ did not show a significant linear relationship with each other (Figure 8) emphasizing that leaf‐level measurements are not the best proxy for iWUE_canopy_. This observation may not hold true in a more water‐limited environment where iWUE_canopy_ and iWUE_leaf_ could be more tightly correlated. Further experiments would be needed to determine how leaf‐level measurements related to the water‐use efficiency can be used as proxies for the overall canopy. Selection of lines within this MAGIC population with high canopy photosynthetic activity will likely contribute to the water‐use efficiency of the canopy, due to the strong linear relationship observed between the two traits.

## 5 CONCLUSION

Canopy architecture was shown to explain a significant part of the variance observed in canopy photosynthesis. Our analysis suggests that 50 MAGIC genotypes can be grouped into 5 general canopy architectural types. In low biomass canopies, the major limitation to canopy photosynthesis is leaf area. However, in higher biomass canopies, the light environment within the canopy became an increasingly limiting factor for canopy photosynthesis. While the negative effects of self‐shading on canopy photosynthesis due to excess leaves have been shown in defoliation experiments (Liu et al., 2015; Srinivasan et al., 2016) and modeling studies (Shuting, 1994), we report a similar response across a cowpea diversity panel. The iWUE_canopy_ in this MAGIC population was mainly explained by canopy photosynthetic activity, and not canopy conductance. Enhancement in canopy photosynthesis is therefore likely to improve the water‐use efficiency of lines from this MAGIC population. Our results provide new insights for breeding program improvement and offer a better understanding of how canopy architecture affects photosynthesis and WUE. As the target environment for cowpea production typically faces water limitation, future analysis could explore how drought alters the relationship between canopy architecture, photosynthesis, WUE, and seed yield. Optimizing canopy architecture under different planting densities also deserves further study.

## Conflict of Interest

None declared.

## Supporting information

Supplemental Information

## References

[fes3236-bib-0001] Ainsworth, E. A. , & Long, S. P. (2005). What have we learned from 15 years of free‐air CO_2_ enrichment (FACE)? A meta‐analytic review of the responses of photosynthesis, canopy properties and plant production to rising CO_2_ . New Phytologist., 165, 351–372. 10.1111/j.1469-8137.2004.01224.x 15720649

[fes3236-bib-0002] Amthor, J. (2000). The McCree‐de Wit‐Penning de Vries‐Thornley respiration paradigms: 30 years later. Annals of Botany, 86, 1–20. 10.1006/anbo.2000.1175

[fes3236-bib-0003] Anyia, A. O. , & Herzog, H. (2004). Water‐use efficiency, leaf area and leaf gas exchange of cowpeas under mid‐season drought. European Journal of Agronomy, 20, 327–339. 10.1016/S1161-0301(03)00038-8

[fes3236-bib-0004] Bailey‐Serres, J. , Parker, J. E. , Ainsworth, E. A. , Oldroyd, G. E. D. , & Schroeder, J. I. (2019). Genetic strategies for improving crop yields. Nature, 575, 109–118. 10.1038/s41586-019-1679-0 31695205 PMC7024682

[fes3236-bib-0005] Bastos, E. A. , Nascimento, S. P. D. , Silva, E. M. D. , Freire Filho, F. R. , & Gomide, R. L. (2011). Identification of cowpea genotypes for drought tolerance. Revista Ciência Agronômica, 42, 100–107. 10.1590/s1806-66902011000100013

[fes3236-bib-0006] Bertolli, S. C. , Rapchan, G. L. , & Souza, G. M. (2012). Photosynthetic limitations caused by different rates of water‐deficit induction in *Glycine max* and *Vigna unguiculata* . Photosynthetica, 50, 329–336. 10.1007/s11099-012-0036-4

[fes3236-bib-0007] Burgess, A. J. , Retkute, R. , Herman, T. , & Murchie, E. H. (2017). Exploring relationships between canopy architecture, light distribution, and photosynthesis in contrasting rice genotypes using 3D canopy reconstruction. Frontiers in Plant Science, 8, 734 10.3389/fpls.2017.00734 28567045 PMC5434157

[fes3236-bib-0008] Burney, J. A. , Naylor, R. L. , & Postel, S. L. (2013). The case for distributed irrigation as a development priority in sub‐Saharan Africa. Proceedings of the National Academy of Sciences, 110, 12513–12517. 10.1073/pnas.1203597110 PMC3732976 23878242

[fes3236-bib-0009] Ciganda, V. , Gitelson, A. , & Schepers, J. (2008). Vertical profile and temporal variation of chlorophyll in maize canopy: Quantitative “crop vigor” indicator by means of reflectance‐based techniques. Agronomy Journal, 100, 1409–1417. 10.2134/agronj2007.0322

[fes3236-bib-0010] Dingkuhn, M. , Singh, B. B. , Clerget, B. , Chantereau, J. , & Sultan, B. (2006). Past, present and future criteria to breed crops for water‐limited environments in West Africa. Agricultural Water Management, 80, 241–261. 10.1016/j.agwat.2005.07.016

[fes3236-bib-0011] Drewry, D. T. , Kumar, P. , Long, S. , Bernacchi, C. , Liang, X.‐Z. , & Sivapalan, M. (2010). Ecohydrological responses of dense canopies to environmental variability: 1. Interplay between vertical structure and photosynthetic pathway. Journal of Geophysical Research, 115, G04022 10.1029/2010JG001340

[fes3236-bib-0012] El Naim, A. M. , & Jabereldar, A. A. (2010). Effect of plant density and cultivar on growth and yield of cowpea (*Vigna unguiculata* L.Walp). Australian Journal of Basic and Applied Sciences, 4, 3148–3153.

[fes3236-bib-0013] FAO (2006). Demand for products of irrigated agriculture in sub‐Saharan Africa. Rome, Italy: FAO.

[fes3236-bib-0014] FAO (2015). Regional overview of food insecurity: African food insecurity prospects brighter than ever. Accra, Ghana: FAO.

[fes3236-bib-0015] FAO (2017). The future of food and agriculture – Trends and challenges. Rome, Italy: FAO.

[fes3236-bib-0016] Fatokun, C. A. , Tarawali, S. A. , Singh, B. B. , Kormawa, P. M. , & Tamò, M. (eds.) (2002). Challenges and opportunities for enhancing sustainable cowpea production. Proceedings of the world cowpea conference III held at the international institute of tropical agriculture (IITA), Ibadan, Nigeria, 4–8 September 2000. International Institute of Tropical Agriculture, Ibadan, Nigeria

[fes3236-bib-0017] Fox, J. , Weisberg, S. , Adler, D. , Bates, D. , Baud‐Bovy, G. , Ellison, S. et al (2016). Car: Companion to applied regression. https://cran.r‐project.org/

[fes3236-bib-0018] Groemping, U. (2006). Relative importance for linear regression in R: The package relaimpo. 10.18637/jss.v017.i01

[fes3236-bib-0019] Hatfield, J. L. , & Dold, C. (2019). Water‐use efficiency: Advances and challenges in a changing climate. Frontiers in Plant Science, 10, 103 10.3389/fpls.2019.00103 30838006 PMC6390371

[fes3236-bib-0020] Huynh, B.‐L. , Ehlers, J. D. , Huang, B. E. , Muñoz‐Amatriaín, M. , Lonardi, S. , Santos, J. R. P. et al (2018). A multi‐parent advanced generation inter‐cross (MAGIC) population for genetic analysis and improvement of cowpea (*Vigna unguiculata* L. Walp.). Plant Journal, 93, 1129–1142. 10.1111/tpj.13827 29356213

[fes3236-bib-0021] Jayathilake, C. , Visvanathan, R. , Deen, A. , Bangamuwage, R. , Jayawardana, B. C. , Nammi, S. , & Liyanage, R. (2018). Cowpea: An overview on its nutritional facts and health benefits. Journal of the Science of Food and Agriculture, 98, 4793–4806. 10.1002/jsfa.9074 29656381

[fes3236-bib-0022] Jemo, M. , Sulieman, S. , Bekkaoui, F. , Olomide, O. A. K. , Hashem, A. , Abd Allah, E. F. et al (2017). Comparative analysis of the combined effects of different water and phosphate levels on growth and biological nitrogen fixation of nine cowpea varieties. Frontiers in Plant Science, 8, 2111 10.3389/fpls.2017.02111 29312379 PMC5742256

[fes3236-bib-0023] Jifon, J. L. , Syvertsen, J. P. , & Whaley, E. (2005). Growth environment and leaf anatomy affect nondestructive estimates of chlorophyll and nitrogen in *Citrus* sp. leaves. Journal of the American Society for Horticultural Science, 130, 152–158. 10.21273/JASHS.130.2.152

[fes3236-bib-0024] Kamara, A. Y. , Ewansiha, S. , Ajeigbe, H. , Omoigui, L. , Tofa, A. I. , & Karim, K. Y. (2017). Agronomic evaluation of cowpea cultivars developed for the West African Savannas. Legume Research: An International Journal, 40, 669–676. 10.18805/lr.v0i0.8410

[fes3236-bib-0025] Koester, R. P. , Skoneczka, J. A. , Cary, T. R. , Diers, B. W. , & Ainsworth, E. A. (2014). Historical gains in soybean (*Glycine max* Merr.) seed yield are driven by linear increases in light interception, energy conversion, and partitioning efficiencies. Journal of Experimental Botany, 65, 3311–3321. 10.1093/jxb/eru187 24790116 PMC4071847

[fes3236-bib-0026] Kong, W. , Huang, W. , Zhou, X. , Ye, H. , Dong, Y. , & Casa, R. (2017). Off‐nadir hyperspectral sensing for estimation of vertical profile of leaf chlorophyll content within wheat canopies. Sensors, 17, 2711 10.3390/s17122711 PMC5751501 29168757

[fes3236-bib-0027] Langyintuo, A. S. , Lowenberg‐DeBoer, J. , Faye, M. , Lambert, D. , Ibro, G. , Moussa, B. , … Ntoukam, G. (2003). Cowpea supply and demand in West and Central Africa. Field Crops Research, 82, 215–231. 10.1016/S0378-4290(03)00039-X

[fes3236-bib-0028] Lê, S. , Josse, J. , & Husson, F. (2008). FactoMineR: an R package for multivariate analysis. Journal of Statistical Software, 25, 1–18. 10.18637/jss.v025.i01

[fes3236-bib-0029] Li, T. , Heuvelink, E. , Dueck, T. A. , Janse, J. , Gort, G. , & Marcelis, L. F. M. (2014). Enhancement of crop photosynthesis by diffuse light: Quantifying the contributing factors. Annals of Botany, 114, 145–156. 10.1093/aob/mcu071 24782436 PMC4071095

[fes3236-bib-0030] Liebsch, D. , & Keech, O. (2016). Dark‐induced leaf senescence: New insights into a complex light‐dependent regulatory pathway. New Phytologist, 212, 563–570. 10.1111/nph.14217 27716940

[fes3236-bib-0031] Liu, G. , Hou, P. , Xie, R. , Ming, B. O. , Wang, K. , Xu, W. , … Li, S. (2017). Canopy characteristics of high‐yield maize with yield potential of 22.5 Mg ha^−1^ . Field Crops Research, 213, 221–230. 10.1016/j.fcr.2017.08.011

[fes3236-bib-0032] Liu, T. , Gu, L. , Dong, S. , Zhang, J. , Liu, P. , & Zhao, B. (2015). Optimum leaf removal increases canopy apparent photosynthesis, ^13^C‐photosynthate distribution and grain yield of maize crops grown at high density. Field Crops Research, 170, 32–39. 10.1016/j.fcr.2014.09.015

[fes3236-bib-0033] Long, S. P. , Zhu, X.‐G. , Naidu, S. L. , & Ort, D. R. (2006). Can improvement in photosynthesis increase crop yields? Plant, Cell & Environment, 29, 315–330. 10.1111/j.1365-3040.2005.01493.x 17080588

[fes3236-bib-0034] Loomis, R. S. , & Williams, W. A. (1969). Productivity and the morphology of crop stands: patterns with leaves In: Eastin J. D., Haskins F. A., Sullivan C. Y., & Van Bavel C. H. M. (Eds.). Physiological aspects of crop yield (pp. 27–47). Madison, WI: American Society of Agronomy 10.2135/1969.physiologicalaspects.c3

[fes3236-bib-0035] Markwell, J. , Osterman, J. C. , & Mitchell, J. L. (1995). Calibration of the Minolta SPAD‐502 leaf chlorophyll meter. Photosynthesis Research, 46, 467–472. 10.1007/BF00032301 24301641

[fes3236-bib-0036] Mateos, L. , & Araus, J. L. (2016). Hydrological, engineering, agronomical, breeding and physiological pathways for the effective and efficient use of water in agriculture. Agricultural Water Management, 164, 190–196. 10.1016/j.agwat.2015.10.017

[fes3236-bib-0037] Morgan, P. B. , Bollero, G. A. , Nelson, R. L. , Dohleman, F. G. , & Long, S. P. (2005). Smaller than predicted increase in aboveground net primary production and yield of field‐grown soybean under fully open‐air [CO_2_] elevation. Global Change Biology, 11, 1856–1865. 10.1111/j.1365-2486.2005.001017.x

[fes3236-bib-0038] Munjonji, L. , Ayisi, K. K. , Boeckx, P. , & Haesaert, G. (2018). Stomatal behavior of cowpea genotypes grown under varying moisture levels. Sustainability, 10, 12 10.3390/su10010012

[fes3236-bib-0039] Murillo‐Amador, B. , Ávila‐Serrano, N. Y. , García‐Hernández, J. L. , López‐Aguilar, R. , Troyo‐Diéguez, E. , & Kaya, C. (2004). Relationship between a nondestructive and an extraction method for measuring chlorophyll contents in cowpea leaves. Journal of Plant Nutrition and Soil Science, 167, 363–364. 10.1002/jpln.200320361

[fes3236-bib-0040] Pepper, G. E. , Pearce, R. B. , & Mock, J. J. (1977). Leaf orientation and yield of maize. Crop Science, 17, 883–886. 10.2135/cropsci1977.0011183X001700060017x

[fes3236-bib-0041] Pérez‐Priego, O. , Testi, L. , Orgaz, F. , & Villalobos, F. J. (2010). A large closed canopy chamber for measuring CO_2_ and water vapour exchange of whole trees. Environmental and Experimental Botany, 68, 131–138. 10.1016/j.envexpbot.2009.10.009

[fes3236-bib-0042] Rivas, R. , Falcão, H. M. , Ribeiro, R. V. , Machado, E. C. , Pimentel, C. , & Santos, M. G. (2016). Drought tolerance in cowpea species is driven by less sensitivity of leaf gas exchange to water deficit and rapid recovery of photosynthesis after rehydration. South African Journal of Botany, 103, 101–107. 10.1016/j.sajb.2015.08.008

[fes3236-bib-0043] Roche, D. (2015). Stomatal conductance is essential for higher yield potential of C3 Crops. Critical Reviews in Plant Sciences, 34, 429–453. 10.1080/07352689.2015.1023677

[fes3236-bib-0044] Rosa, L. , Rulli, M. C. , Davis, K. F. , Chiarelli, D. D. , Passera, C. , & D’Odorico, P. (2018). Closing the yield gap while ensuring water sustainability. Environmental Research Letters, 13, 104002 10.1088/1748-9326/aadeef

[fes3236-bib-0045] RStudio team (2015). RStudio: integrated development for R. Boston, MA: RStudio, Inc http://www.rstudio.com

[fes3236-bib-0046] Ryan, M. G. (1991). Effects of climate change on plant respiration. Ecological Applications, 1, 157–167. 10.2307/1941808 27755662

[fes3236-bib-0047] Sanz‐Sáez, Á. , Koester, R. P. , Rosenthal, D. M. , Montes, C. M. , Ort, D. R. , & Ainsworth, E. A. (2017). Leaf and canopy scale drivers of genotypic variation in soybean response to elevated carbon dioxide concentration. Global Change Biology, 23, 3908–3920. 10.1111/gcb.13678 28267246

[fes3236-bib-0048] Sennhenn, A. , Njarui, D. M. G. , Maass, B. L. , & Whitbread, A. M. (2017). Exploring niches for short‐season grain legumes in semi‐arid eastern kenya ‐ coping with the impacts of climate variability. Frontiers in Plant Science, 8, 699 10.3389/fpls.2017.00699 28536585 PMC5422554

[fes3236-bib-0049] Sheehy, J. E. , & Mitchell, P. L. (2013). Designing rice for the 21st century: the three laws of maximum yield. Discussion Paper Series 48. Los Baños (Philippines): International Rice Research Institute.

[fes3236-bib-0050] Shuting, D. (1994). Canopy apparent photosynthesis, respiration and yield in wheat. The Journal of Agricultural Science, 122, 7–12. 10.1017/S0021859600065722

[fes3236-bib-0051] Simkin, A. J. , López‐Calcagno, P. E. , & Raines, C. A. (2019). Feeding the world: Improving photosynthetic efficiency for sustainable crop production. Journal of Experimental Botany, 70, 1119–1140. 10.1093/jxb/ery445 30772919 PMC6395887

[fes3236-bib-0052] Sinclair, T. R. , & Muchow, R. C. (1999). Radiation use efficiency. Advance in Agrononomy, 65, 215–265. 10.1016/S0065-2113(08)60914-1

[fes3236-bib-0053] Sinclair, T. R. , & Sheehy, J. E. (1999). Erect leaves and photosynthesis in rice. Science, 283, 1455 10.1126/science.283.5407.1455c

[fes3236-bib-0054] Singh, B. B. , Ajeigbe, H. A. , Tarawali, S. A. , Fernandez‐Rivera, S. , & Abubakar, M. (2003). Improving the production and utilization of cowpea as food and fodder. Field Crops Research, 84, 169–177. 10.1016/S0378-4290(03)00148-5

[fes3236-bib-0055] Singh, S. K. , & Raja Reddy, K. (2011). Regulation of photosynthesis, fluorescence, stomatal conductance and water‐use efficiency of cowpea (*Vigna unguiculata* [L.] Walp.) under drought. Journal of Photochemistry and Photobiology B: Biology, 105, 40–50. 10.1016/j.jphotobiol.2011.07.001 21820316

[fes3236-bib-0056] Song, Q. , Srinivasan, V. , Long, S. P. , & Zhu, X.‐G. (2019). Decomposition analysis on soybean productivity increase under elevated CO_2_ using 3D canopy model reveals synergestic effects of CO_2_ and light in photosynthesis. Annals of Botany, mcz163, 1–14. 10.1093/aob/mcz163 PMC7489077 31638642

[fes3236-bib-0057] Song, Q. , Wang, Y. , Qu, M. , Ort, D. R. , & Zhu, X.‐G. (2017). The impact of modifying photosystem antenna size on canopy photosynthetic efficiency – Development of a new canopy photosynthesis model scaling from metabolism to canopy level processes. Plant, Cell & Environment, 40, 2946–2957. 10.1111/pce.13041 PMC5724688 28755407

[fes3236-bib-0058] Song, Q. , Zhang, G. , & Zhu, X.‐G. (2013). Optimal crop canopy architecture to maximise canopy photosynthetic CO_2_ uptake under elevated CO_2_ – a theoretical study using a mechanistic model of canopy photosynthesis. Functional Plant Biology, 40, 108–124. 10.1071/FP12056 32481092

[fes3236-bib-0059] Song, Q. , & Zhu, X.‐G. (2018). Measuring canopy gas exchange using CAnopy Photosynthesis and Transpiration Systems (CAPTS) In: Covshoff S. (Eds.) Photosynthesis. Methods in molecular biology, vol 1770. New York, NY: Humana Press. 10.1007/978-1-4939-7786-4_4 29978396

[fes3236-bib-0060] Sprent, J. I. , Odee, D. W. , & Dakora, F. D. (2009). African legumes: A vital but under‐utilized resource. Journal of Experimental Botany, 61, 1257–1265. 10.1093/jxb/erp342 19939887

[fes3236-bib-0061] Srinivasan, V. , Kumar, P. , & Long, S. P. (2016). Decreasing, not increasing, leaf area will raise crop yields under global atmospheric change. Global Change Biology, 23, 1626–1635. 10.1111/gcb.13526 27860122 PMC5347850

[fes3236-bib-0062] Steduto, P. , Çetinkökü, Ö. , Albrizio, R. , & Kanber, R. (2002). Automated closed‐system canopy‐chamber for continuous field‐crop monitoring of CO_2_ and H_2_O fluxes. Agricultural and Forest Meteorology, 111, 171–186. 10.1016/S0168-1923(02)00023-0

[fes3236-bib-0063] Tarawali, S. A. , Okike, I. , Kristjanson, P. K. , Singh, B. B. , & Thornton, P. (2005). Dual‐purpose cowpea for west Africa. Tropical Grasslands, 39, 210.

[fes3236-bib-0064] Torres Netto, A. , Campostrini, E. , De Oliveira, J. G. , & Bressan‐Smith, R. E. (2005). Photosynthetic pigments, nitrogen, chlorophyll a fluorescence and SPAD‐502 readings in coffee leaves. Scientia Horticulturae, 104, 199–209. 10.1016/j.scienta.2004.08.013

[fes3236-bib-0065] USDA National Agricultural Statistics Service (2019). Crop production 2018 summary (February 2019).

[fes3236-bib-0066] van lttersum, M. K. , van Bussel, L. G. J. , Wolf, J. , Grassini, P. , van Wart, J. , Guilpart, N. , Cassman, K. G. (2016) Can sub‐Saharan Africa feed itself? Proceedings of the National Academy of Sciences, 113(52), 14964–14969. 10.1073/pnas.1610359113 PMC5206509 27956604

[fes3236-bib-0067] Waddington, S. R. , Li, X. , Dixon, J. , Hyman, G. , & de Vicente, M. C. (2010). Getting the focus right: Production constraints for six major food crops in Asian and African farming systems. Food Security, 2, 27–48. 10.1007/s12571-010-0053-8

[fes3236-bib-0068] Walker, B. J. , Drewry, D. T. , Slattery, R. A. , VanLoocke, A. , Cho, Y. B. , & Ort, D. R. (2017). Chlorophyll can be reduced in crop canopies with little penalty to photosynthesis. Plant Physiology, 176, 1215–1232. 10.1104/pp.17.01401 29061904 PMC5813550

[fes3236-bib-0069] Wang, Q. , Chen, J. , & Li, Y. (2004). Nondestructive and rapid estimation of leaf chlorophyll and nitrogen status of peace lily using a chlorophyll meter. Journal of Plant Nutrition, 27, 557–569. 10.1081/PLN-120028878

[fes3236-bib-0070] Wang, Y.‐Z. , Hong, W. , Wu, C.‐Z. , Lin, H. , Fan, H.‐L. , Chen, C. , & Li, J. (2009). Variation of SPAD values in uneven‐aged leaves of different dominant species in *Castanopsis carlessi* forest in Lingshishan National Forest Park. Journal of Forestry Research, 20, 362–366. 10.1007/s11676-009-0061-8

[fes3236-bib-0071] Weber, A. P. M. , & Bar‐Even, A. (2019). Update: Improving the efficiency of photosynthetic carbon reactions. Plant Physiology, 179, 803–812. 10.1104/pp.18.01521 30610109 PMC6393813

[fes3236-bib-0072] Winterhalter, L. , Mistele, B. , & Schmidhalter, U. (2012). Assessing the vertical footprint of reflectance measurements to characterize nitrogen uptake and biomass distribution in maize canopies. Field Crops Research, 129, 14–20. 10.1016/j.fcr.2012.01.007

[fes3236-bib-0073] Wu, A. , Hammer, G. L. , Doherty, A. , von Caemmerer, S. , & Farquhar, G. D. (2019). Quantifying impacts of enhancing photosynthesis on crop yield. Nature Plants, 5, 380–388. 10.1038/s41477-019-0398-8 30962528

[fes3236-bib-0074] Zhu, X.‐G. , Long, S. P. , & Ort, D. R. (2010). Improving photosynthetic efficiency for greater yield. Annual Review of Plant Biology, 61, 235–261. 10.1146/annurev-arplant-042809-112206 20192734

