# Supplementary material for: Assessing diversity in canopy architecture, photosynthesis, and water‐use efficiency in a cowpea magic population
Source: Food Energy Secur. 2020 Aug 7;9(4):e236. doi: 10.1002/fes3.236 (PMC7757253; doi:10.1002/fes3.236)
Supplement: Supplementary file 1 — Supplemental Information [file FES3-9-e236-s001.docx]

# Supplementary materials

| Document | Full_dataset.txt |
| --- | --- |

Supp. material 1. List of genotypes and values for all traits.


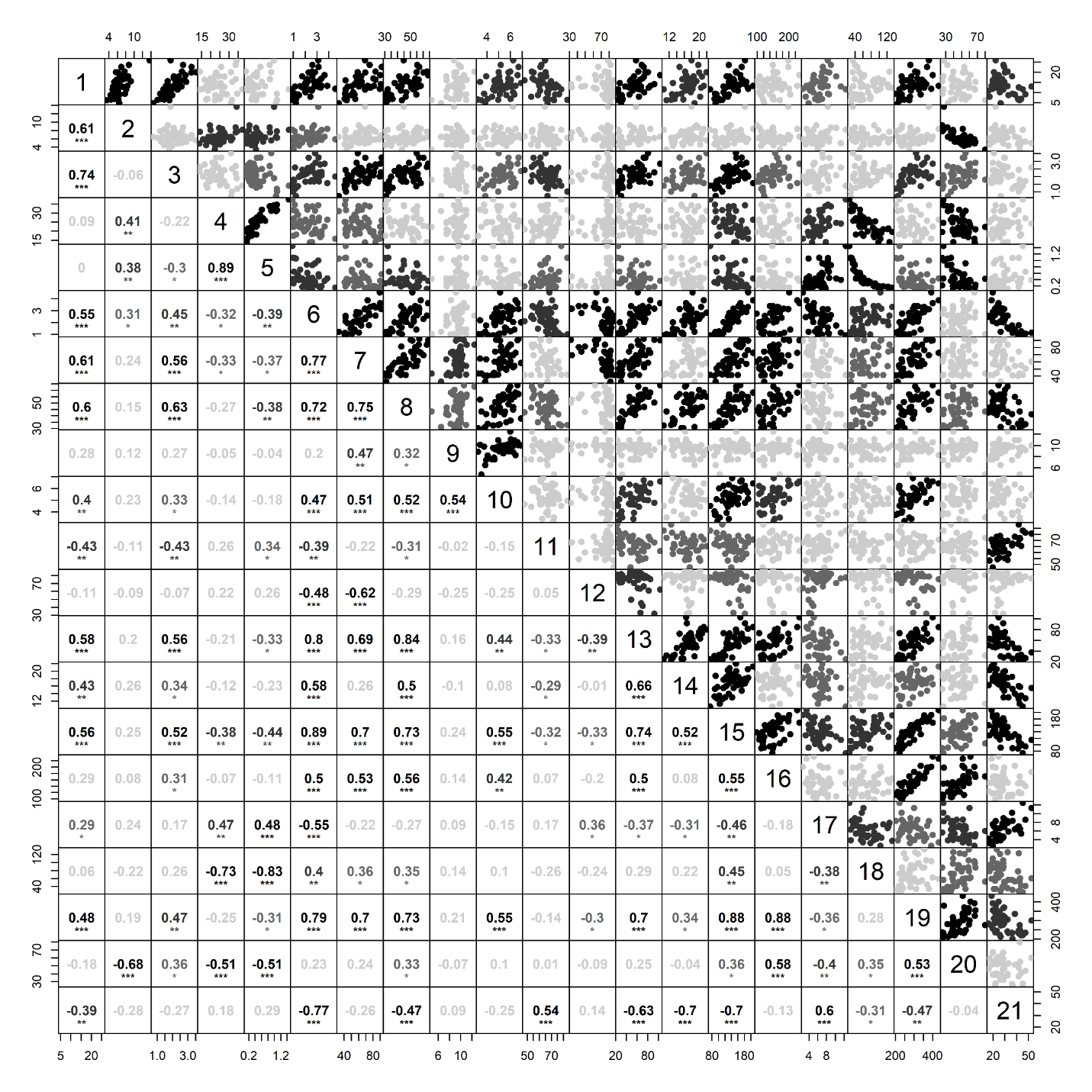


Supp. Material 2. Pearson correlation matrix. 1. *A_canopy,ground_*; 2. *g_c_*; 3. iWUE_canopy_; 4. A*_leaf_*; 5. *g_s_*; 6. leaf area; 7. canopy width; 8. canopy height; 9. leaf length; 10. leaf width; 11. spad; 12. stem angle; 13. stem length; 14. nodes number; 15. leaf mass; 16. shoot mass; 17. *A_canopy,LA_*; 18. iWUE_leaf_; 19. total biomass; 20. WUE_biomass_; 21. leaf area exposure. ns, *, **, and *** indicate a >0.05, <0.05, <0.01, and <0.001 p-value for the correlation test.

Supp. Material 3. Correlation of variables with principal components (PC) for the PCA-HCPC used to determine clusters of cowpea lines with contrasting architectures.

|  | PC1 | PC 2 | PC 3 |
| --- | --- | --- | --- |
| Canopy width | 0.823416 | 0.374159 | -0.00443 |
| Canopy height | 0.892808 | 0.023071 | 0.09536 |
| Leaf length | 0.307987 | 0.658986 | -0.46894 |
| Leaf width | 0.60413 | 0.438654 | -0.20908 |
| SPAD | -0.40862 | 0.428764 | 0.615612 |
| Stem angle | -0.442 | -0.38701 | 0.11425 |
| Stem length | 0.907407 | -0.14674 | 0.134516 |
| Nodes | 0.606013 | -0.61591 | 0.10329 |
| Leaf mass | 0.904433 | -0.07106 | 0.073423 |
| Shoot mass | 0.595887 | 0.302216 | 0.579187 |
| Leaf area exposure | -0.66974 | 0.600123 | 0.156623 |

.


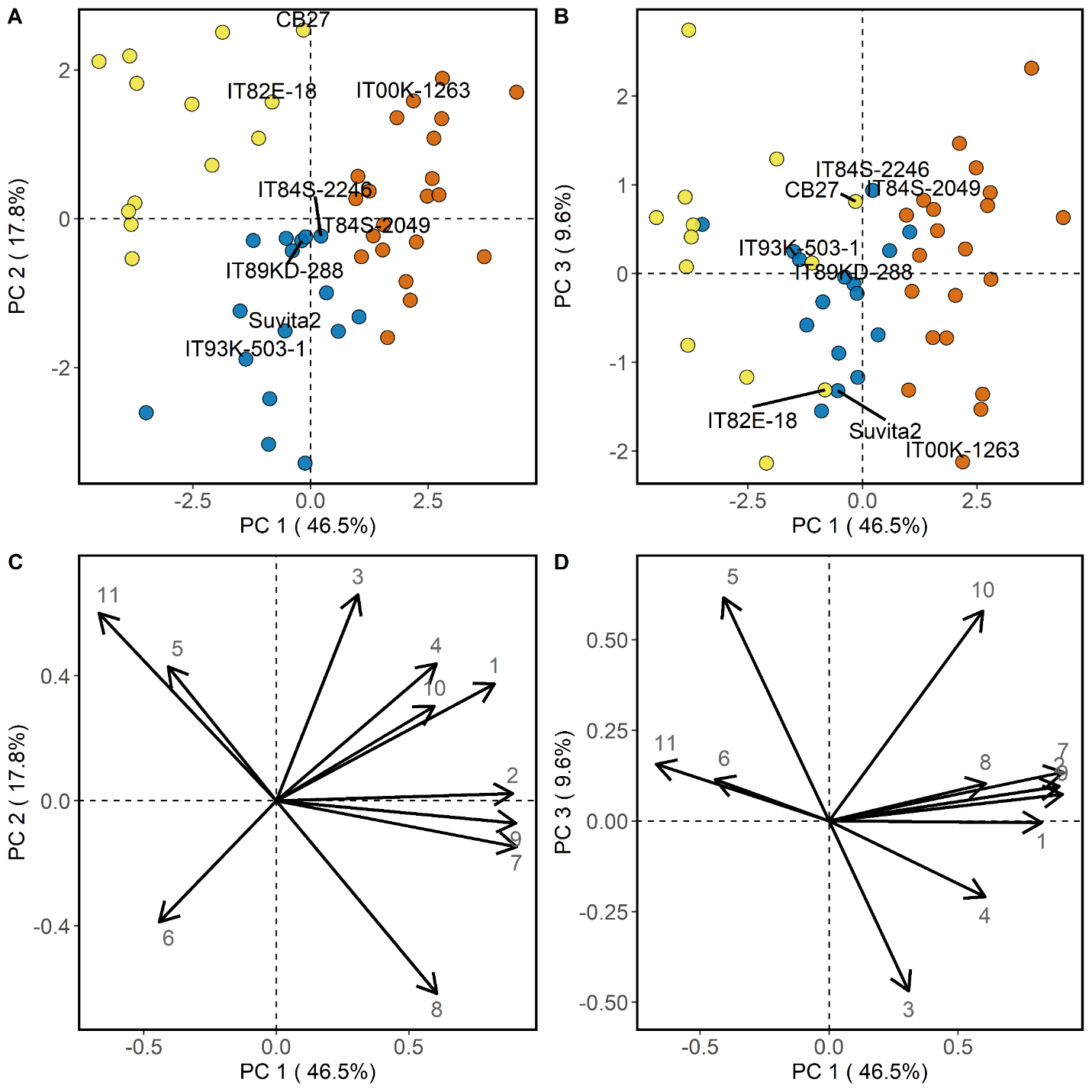


Supp. Material 4. Definition of three clusters with similar and contrasting canopy features. Determination of clusters with contrasting phenotypes was obtained by principal component analysis (PCA) followed by clustering. Distribution according to the principal components 1-2 (A) and 1-3 (B) and the correlation circles with the variables loaded on the principal components 1-2 (C) and 1-3 (D) are represented. The clusters are highlighted by different colors with clusters 1, 2, and 3 shown in yellow, blue, and red. Canopy traits entered as variables in the PCA were: 1 – canopy width; 2 – canopy height; 3 – leaf length; 4 – leaf width; 5 – SPAD; 6 – stem angle; 7 – stem length; 8 – nodes; 9 – leaf mass; 10 – shoot mass; 11 – leaf area exposure.

Supp. Material 5. Average (± SD) for each trait within the different clusters defined in Supp. Material 4. Significant p-value indicates significant differences among clusters. ns, *, **, and *** indicate a >0.05, <0.05, <0.01, and <0.001 p-value when tested with a linear model. Different letters indicate significant differences among the clusters (Tukeys’ HSD, ɑ= 0.05).

| Traits | Cluster 1 (n=13) | Cluster 2 (n=16) | Cluster 3 (n=21) | P-values |
| --- | --- | --- | --- | --- |
| *A_leaf_* | 25.89 + 5.97 a | 24.88 + 4.87 a | 22.4 + 6.15 a | ns |
| *g_s_* | 0.58 + 0.39 a | 0.44 + 0.28 a | 0.39 + 0.27 a | ns |
| iWUE_leaf_ | 57.39 + 27.14 a | 71.05 + 27.39 a | 75.42 + 30.21 a | ns |
| *A_canopy,ground_* | 9.29 + 2.82 c | 13.43 + 3.83 b | 16.57 + 4.33 a | *** |
| *A_canopy,LA_* | 7.58 + 2.11 a | 6.42 + 1.87 ab | 5.52 + 1.89 b | * |
| *g_c_* | 5.61 + 1.35 b | 6.67 + 1.17 ab | 7 + 1.94 a | ns |
| iWUE_canopy_ | 1.7 + 0.51 b | 2.01 + 0.48 ab | 2.42 + 0.56 a | ** |
| iWUE_biomass_ | 47.64 + 14.04 ab | 40.51 + 9.34 b | 51.14 + 11.85 a | * |
| SPAD | 71.1 + 6.64 a | 63.6 + 6.79 b | 63.53 + 6.07 b | ** |
| Leaf length | 9.35 + 1.56 a | 8.64 + 1.22 a | 9.75 + 0.98 a | ns |
| Leaf width | 4.94 + 1.12 b | 4.73 + 0.71 b | 5.75 + 0.55 a | ** |
| Leaf area | 1.25 + 0.33 c | 2.12 + 0.41 b | 3.11 + 0.53 a | *** |
| Leaf area exposure | 41.35 + 6.13 a | 25.15 + 5.65 b | 24.76 + 4.03 b | *** |
| Stem angle | 76.7 + 3.08 a | 80.78 + 4.11 a | 64.69 + 16.85 b | *** |
| Stem length | 31.6 + 7.38 c | 53.7 + 11.01 b | 71.04 + 12.87 a | *** |
| Nodes | 13.56 + 1.7 b | 18.23 + 1.44 a | 18.04 + 1.87 a | *** |
| Canopy width | 50.43 + 9.9 b | 51.93 + 10.13 b | 75.89 + 10.79 a | *** |
| Canopy height | 37.85 + 7.7 c | 44.47 + 5.29 b | 54.47 + 5.37 a | *** |
| Leaf mass | 107.23 + 19.37 c | 134.5 + 17.96 b | 167.37 + 20.19 a | *** |
| Shoot mass | 145 + 28.8 b | 127 + 20.28 b | 173.11 + 27.27 a | *** |
| Total biomass | 252.23 + 38.16 b | 261.5 + 30.73 b | 340.47 + 43 a | *** |

| Clusters | Traits |  | *A_canopy,ground_* |  |  | *A_canopy,LA_* |  |
| --- | --- | --- | --- | --- | --- | --- | --- |
|  |  | **Slopes** | **R^2^** | **P-values** | **Slopes** | **R^2^** | **P-values** |
| 1 | *A_canopy,ground_* | - | - | - | 0.422 | 0.317 | * |
| (n=13) | *A_canopy,LA_* | 0.75 | 0.317 | * | - | - | - |
|  | iWUE_canopy_ | 4.04 | 0.526 | ** | 0.94 | 0.05 | ns |
|  | *A_leaf_* | 0.099 | 0.044 | ns | 0.201 | 0.324 | * |
|  | Leaf area | 5.379 | 0.408 | * | -1.7 | 0.072 | ns |
|  | Canopy width | 0.18 | 0.399 | * | -0.003 | 0 | ns |
| 2 | *A_canopy,ground_* | - | - | - | 0.341 | 0.532 | ** |
| (n=16) | *A_canopy,LA_* | 1.561 | 0.532 | ** | - | - | - |
|  | *g_c_* | 1.882 | 0.328 | * | 0.785 | 0.239 | ns |
|  | iWUE_canopy_ | 6.494 | 0.669 | *** | 2.445 | 0.397 | * |
|  | Leaf mass | 0.119 | 0.323 | * | 0.016 | 0.027 | ns |
|  | Leaf area exposure | 0.139 | 0.041 | ns | 0.228 | 0.506 | ** |
|  | Canopy width | 0.303 | 0.618 | *** | 0.102 | 0.318 | * |
|  | Canopy height | 0.343 | 0.264 | * | 0.027 | 0.008 | ns |
|  | Stem angle | -0.449 | 0.216 | ns | -0.23 | 0.259 | * |
| 3 | *A_canopy,ground_* | - | - | - | 0.388 | 0.837 | 0 |
| (n=21) | *A_canopy,LA_* | 2.157 | 0.837 | 0 | - | - | - |
|  | *g_c_* | 1.346 | 0.365 | ** | 0.411 | 0.178 | ns |
|  | iWUE_canopy_ | 3.905 | 0.252 | * | 1.96 | 0.334 | ** |
|  | iWUE_biomass_ | -0.194 | 0.282 | * | -0.081 | 0.258 | * |
|  | *A_leaf_* | 0.471 | 0.457 | *** | 0.169 | 0.328 | ** |
|  | *g_s_* | 10.472 | 0.589 | 0 | 3.936 | 0.463 | *** |
|  | iWUE_leaf_ | -0.089 | 0.391 | ** | -0.034 | 0.317 | ** |
|  | SPAD | -0.344 | 0.195 | * | -0.156 | 0.223 | * |
|  | Leaf area | -2.963 | 0.111 | ns | -2.46 | 0.424 | ** |
|  | Leaf area exposure | 0.349 | 0.086 | ns | 0.246 | 0.239 | * |
|  | Stem angle | 0.105 | 0.151 | ns | 0.055 | 0.232 | * |

Supp. Material 6. Linear regression of *A_canopy_* with other traits within each cluster. Only traits with a p-value < 0.05 for the linear regression are shown. ns, *, **, and *** indicate a >0.05, <0.05, <0.01, and <0.001 p- value for the linear regression. As for the case where 5 clusters were defined, leaf area showed a positive linear relationship with *A_canopy,ground_* in the cluster of genotypes characterized by lower leaf area. Increase of canopy width for this cluster did not contribute to increased *A_canopy,LA_*. For the cluster 3, characterized by high biomass and leaf area, SPAD showed a negative linear relationship with *A_canopy_*. Leaf area exposure contributed positively to *A_canopy,LA_* but not to *A_canopy,ground_*. *A_leaf_* explained a greater proportion (45.7%) of the variation in *A_canopy,ground_*_,_ likely because of greater variation in *A*_leaf_ within the cluster.


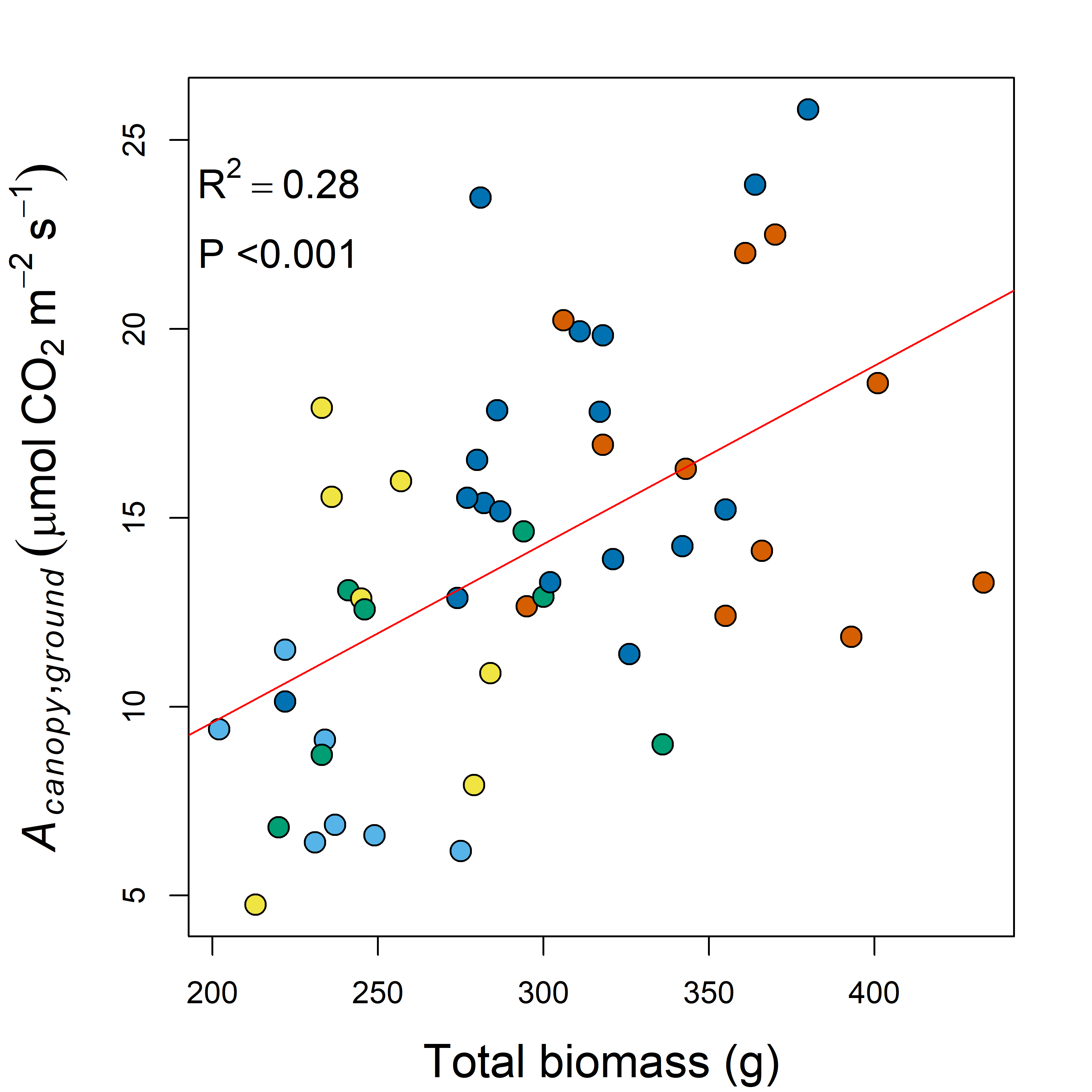


Supp. Material 7. Linear regression between *A_canopy,ground_* and total biomass. Genotypes are colored based on clustering (Fig. 5) with clusters 1, 2, 3, 4 and 5 shown in light blue, green, yellow, dark blue and red.
